# Selection of scFv Antibody Fragments Binding to Human Blood versus Lymphatic Endothelial Surface Antigens by Direct Cell Phage Display

**DOI:** 10.1371/journal.pone.0127169

**Published:** 2015-05-20

**Authors:** Thomas Keller, Romana Kalt, Ingrid Raab, Helga Schachner, Corina Mayrhofer, Dontscho Kerjaschki, Brigitte Hantusch

**Affiliations:** 1 Clinical Institute of Pathology, Medical University of Vienna, Vienna, Austria; 2 Institute for Animal Breeding and Genetics, University of Veterinary Medicine, Vienna, Austria; Naval Research Laboratory, UNITED STATES

## Abstract

The identification of marker molecules specific for blood and lymphatic endothelium may provide new diagnostic tools and identify new targets for therapy of immune, microvascular and cancerous diseases. Here, we used a phage display library expressing human randomized single-chain Fv (scFv) antibodies for direct panning against live cultures of blood (BECs) and lymphatic (LECs) endothelial cells in solution. After six panning rounds, out of 944 sequenced antibody clones, we retrieved 166 unique/diverse scFv fragments, as indicated by the V-region sequences. Specificities of these phage clone antibodies for respective compartments were individually tested by direct cell ELISA, indicating that mainly pan-endothelial cell (EC) binders had been selected, but also revealing a subset of BEC-specific scFv antibodies. The specific staining pattern was recapitulated by twelve phage-independently expressed scFv antibodies. Binding capacity to BECs and LECs and differential staining of BEC versus LEC by a subset of eight scFv antibodies was confirmed by immunofluorescence staining. As one antigen, CD146 was identified by immunoprecipitation with phage-independent scFv fragment. This antibody, B6-11, specifically bound to recombinant CD146, and to native CD146 expressed by BECs, melanoma cells and blood vessels. Further, binding capacity of B6-11 to CD146 was fully retained after fusion to a mouse Fc portion, which enabled eukaryotic cell expression. Beyond visualization and diagnosis, this antibody might be used as a functional tool. Overall, our approach provided a method to select antibodies specific for endothelial surface determinants in their native configuration. We successfully selected antibodies that bind to antigens expressed on the human endothelial cell surfaces *in situ*, showing that BECs and LECs share a majority of surface antigens, which is complemented by cell-type specific, unique markers.

## Introduction

The microvasculature is a specialized compartment consisting of blood and lymphatic vessels. Blood (BEC) and lymphatic endothelial cells (LEC), which are lining these two separate vascular systems, have been recognized as functionally distinct cell lineages showing characteristic profiles of gene expression. However, until now, only a few markers are accepted as highly lineage-specific markers, including PAL-E or MECA-32 for BECs [1,2], and podoplanin or LYVE-1, for LECs [3,4]. Using these markers, it has become possible to isolate primary cultures of BEC and LEC subpopulations [5], which maintain their characteristic features over several passages, and several transcriptomal analyses thereof have been performed [6]. The data suggest a high similarity between these two cell lines, but also point at a cell-type specific molecular repertoire. However, transcriptomic data may not directly refer to the protein expression levels present on the cell surface and, still, markers that are characteristic for endothelial specialization at different anatomic locations are barely known. Further, small vessels are associated with prevalent disorders such as cardiovascular diseases, diabetes and cancer, in which they undergo unphysiological cellular transformation. Molecules that are altered are likely to have functional roles that participate in disease progress, and might have therapeutic value. Hence, it is highly important to derive information about the native abundance and antigenic properties of BEC and LEC surface molecules *ex vivo*, in order to reliably trace alterations thereof.

During the last decade, we have undertaken several strategies to characterize BEC and LEC subcompartments more detailed [5,7], and we were successful in defining some key aspects of alterations on transcriptomal level which provided us with approximate patterns of gene activity [8,9]. In order to understand more about the molecular repertoire of BEC and LEC lineages, here, we aimed to identify their antigenic features in a more straight-forward approach. Immunization and subsequent hybridoma technologies are time-consuming and have technical limitations. Therefore, we turned to the use of phage display of a scFv antibody library to enrich for phage antibodies that would be highly specific for both and / or discriminative between the two cell line surfaces.

The isolation of monoclonal antibodies to specific antigenic targets has been revolutionized by antibody phage display technology [10], which allows for rapid selection and isolation of antibodies to a given target antigen [11]. Phage display has been used to generate antibodies targeting a plethora of antigens [12,13,14,15], showing the usefulness of this methodology. Moreover, phage display has been successfully used to isolate antibodies to more complex sources such as known cell-surface expressed antigens [16,17,18], but also on endothelial cell lines [19,20,21,22], and even vascular tissues *in vivo* [23,24,25,26], without previous knowledge of the antigens.

The antibody repertoire displayed by phage particles can be either natural, immunized or non-immune [11,27,28]. Our source of antibody genes was the ETH-2 library, a semi-synthetic phage library displaying human single chain Fv antibodies (scFvs) [29] that contains randomized sequences based on one gene for the heavy, and two genes for the light chains. ETH-2 was previously successfully applied to isolate antibodies against endothelial antigens [29,30,31]. This is the first report of its use to perform screenings on primary human BECs and LECs to generate a catalogue of antibodies against membrane-associated proteins, showing that endothelial cells can be directly used as antigen carriers.

## Materials and Methods

### Sorting and culturing of blood and lymphatic endothelial cell lines

Human dermal microvascular endothelial cells (HDMECs), derived from human foreskin, were purchased from Promocell (no. C-12260). This cell line is generated in strict compliance with ethical and legal standards (http://www.promocell.com/products/ethical-standards/) and has been published previously in numerous articles (http://www.promocell.com/knowledge-base/search-for-information/) [9].

Separation of podoplanin-positive LECs from BECs was performed by using magnetic beads (Dynal) coated with anti-podoplanin antibody, as described previously [5]. Cells were grown in endothelial basal medium (EBM-2) supplemented with 5% fetal calf serum (FCS) and EGM-2-MV SingleQuots (CC-4147; Lonza) in an incubator at 37°C and 5% CO2. For all experiments, LECs and BECs were used below passage 6.

### Immunofluorescence stainings

For cell surface immunofluorescence, cells were grown on Lab-Tek II Chamber Slides (Nunc) and fixed with 1% PFA in PBS. For tissue immunohistochemistry, human dermal biopsies were embedded in OCT medium, frozen, cut into 5μm sections at -20°C and used immediately, or stored at -20 to -80°C. The study was approved by the local ethics committee (Proposal no. 1228/2014). For immunofluorescence stainings, slides were thawed, dried at room temperature for 10 min and fixed in ice-cold acetone or in PBS/ 1% PFA for 20 min. Blocking of sections was performed for 30 min at RT in PBS/ 10% goat or donkey serum, depending on the applied antibodies (S1 Table). Slides were washed briefly in PBS, and primary antibody was added for 1 hr at RT or overnight at 4°C, depending on the antibody. Cells were incubated with scFv antibodies diluted 1:50 in PBS/ 2% BSA and, as controls, with secondary antibody alone or with unspecific scFv. Slides were washed 3 times for 5 min in PBS and the secondary antibody dilution was applied (FITC-labeled anti-flag tag antibody or fluorescence labeled secondary antibodies diluted in PBS/ 2% serum for blocking). Nuclei were counterstained with DAPI and slides were mounted with Geltol. Pictures were taken with a VANOX AHBT3 fluorescence microscope (Olympus) an inverted live cell microscope (AxioVert 200M, Zeiss), or a laser scanning microscope (LSM 5 Exciter, Zeiss).

### scFv phage display library

The phage antibody library ETH-2, a non-immunised single chain antibody fragment (scFv) library of over 5 x 10^8^ clones [29], was used for selections on BECs and LECs. ETH-2 phages display human recombinant scFv antibodies, in which one human germline gene segment for the heavy and two germline gene segments for the light chains [32] were used together with randomized positions in both complement determining region 3 (CDR3) loops [33]. The library was cloned into restriction sites *Nco*I and *Not*I of phagemid vector pDN332, carrying an ampicillin resistance, a lacZ promoter, a pelB leader sequence for secretion of scFv fragments into culture supernatants, a His-tag and a Flag-tag applicable for detection.

### Selection of phages on BECs and LECs

Six rounds of selection were performed on BECs and LECs with the ETH-2 scFv library, essentially according to recommended protocol [29], with changes due to panning procedure on cells. Selections were carried out using detached cells rather than confluent monolayers, as panning on these yielded mainly unspecific phage antibodies (own communication). Briefly, BECs and LECs nearly confluently grown in T75 cell culture flasks were washed with PBS and mildely detached with 5mM EDTA/ PBS at 37°C. Cells were harvested after 15–30 min and centrifuged at 1500 rpm for 3 min. Cell pellets were weighed and resuspended in 1 ml EGM-2 MV/ 1% BSA. The solution was split into two 500μl portions containing approximately 1 x 10^5^ cells. To each tube, 200 μl of ETH-2 library (~ 10^12^ pfu/ml) or VCS-M13 helper phage (~ 10^12^ pfu/ml) as background control were added (S2 Fig), respectively, and tubes were incubated overnight at 4°C under rotation. On day 2, cells were centrifuged at 1.500 rpm for 3 min and resuspended in 1 ml EGM2-MV/ 1% BSA. Cell suspensions were transferred to new 1.5ml tubes and unbound and weak bound phages were removed by washing the cells four times with EGM2-MV/ 1% BSA, finally resuspending them in 400 μl PBS. Phage-carrying cells were directly used for re-infection of 7.5 ml exponentially growing TG1 *E*. *coli* culture. After 30 min at 37°C, 25 μl of infected TG1 bacteria were used for determination of phage output titer. Serial 1:100 dilutions (10^–5^, 10^–7^, 10^–9^, 10^–11^) were prepared in 2xTY medium, and 10 μl (titer = colonies x 1.000) of each dilution were plated on 2xTY-Amp-Glu plates, or on 2xTY-Kana-Glu plates for helper phage infected bacteria. To amplify the whole phagemid DNA pool, remaining infected TG1 were centrifuged at 3.300 g for 10 min, pellets were resuspended in 0.5 ml 2xTY, spread on 500 cm^2^ 2xTY-Amp-Glu agar culture dishes and incubated at 30°C overnight. On day 3, TG1 bacteria grown on the large agar plates were gently loosened by adding 5–10 ml 2xTY/ 10% glycerol with a glass spreader. The homogenous bacterial suspensions were aspirated and stored at—80°C, or immediately used for phage particle preparation. Colonies of plates with dilution series were counted and phage titers were calculated, yielding the output titer/ml (1–5 x 10^12^ pfu/ml per round). Titers of input and output phage were corrected for different volumes and expressed as the total amount of cfu. Phage recovery was calculated as the ratio of output/input phage after one round of selection. Enrichment factors were calculated as output versus input ratios as previously described [17] (Table 1).

**Table 1:** Phage recoveries and enrichment factors during selection of ETH2 phage library on BECs and LECs in suspension.

Selection round	Phage source	Cells	Input [cfu] ^a^	Input ratio ^b^	Output [cfu/g] ^c^	Output ratio ^d^	Recovery[%] ^e^	Enrichment factor ^f^
**#1**	ETH2	BEC	4.0 x 10^11^	2.0	1.2 x 10^9^	83	0.31	**42**
	WT	BEC	2.0 x 10^11^		1.5 x 10^7^		0.01	
	ETH2	LEC	8.1 x 10^11^	2.0	3.7 x 10^9^	49	0.45	**24**
	WT	LEC	4.0 x 10^11^		7.5 x 10^7^		0.02	
**#2**	ETH2	BEC	1.2 x 10^12^	6.2	3.4 x 10^10^	8509	2.72	**1378**
	WT	BEC	2.0 x 10^11^		3.9 x 10^6^		0.002	
	ETH2	LEC	1.6 x 10^12^	8.0	2.0 x 10^10^	1084	1.26	**135**
	WT	LEC	2.0 x 10^11^		1.9 x 10^7^		0.01	
**#3**	ETH2	BEC	6.7 x 10^11^	3.4	4.9 x 10^10^	190	7.40	**56**
	WT	BEC	2.0 x 10^11^		2.6 x 10^8^		0.13	
	ETH2	LEC	5.2 x 10^11^	2.6	2.8 x 10^10^	1934	5.41	**744**
	WT	LEC	2.0 x 10^11^		1.5 x 10^7^		0.01	
**#4**	ETH2	BEC	2.1 x 10^10^	0.1	4.8 x 10^9^	186	22.74	**1774**
	WT	BEC	2.0 x 10^11^		2.6 x 10^7^		0.01	
	ETH2	LEC	4.0 x 10^10^	0.2	6.5 x 10^9^	5785	16.16	**28926**
	WT	LEC	2.0 x 10^11^		1.1 x 10^6^		0.001	
**#5**	ETH2	BEC	2.1 x 10^11^	1.0	9.9 x 10^10^	578	47.81	**558**
	WT	BEC	2.0 x 10^11^		1.7 x 10^8^		0.09	
	ETH2	LEC	1.2 x 10^11^	0.6	7.8 x 10^10^	3533	64.71	**5752**
	WT	LEC	2.0 x 10^11^		2.2 x 10^7^		0.01	
**#6**	ETH2	BEC	7.0 x 10^10^	0.2	5.7 x 10^10^	8163	82.10	**40816**
	WT	BEC	3.5 x 10^11^		7.0 x 10^6^		0.002	
	ETH2	LEC	1.5 x 10^11^	0.3	1.0 x 10^11^	1117	69.52	**4251**
	WT	LEC	5.9 x 10^11^		9.3 x 10^7^		0.02	

Selection was performed separately on BECs and LECs with ETH2 library and WT phage as control.

^a^ Input phage amount in absolute CFU

^b^ Numbers represent Input (ETH2) / Input (WT) phage ratios

^c^ Output phage amount normalized to 1g of cell pellet

^d^ Numbers represent Output (ETH2) / Output (WT) phage ratio

^e^ Recovery is given as Output / Input

^f^ Enrichment factor is given as Output ratio / Input ratio.

Phage rescue from each selection round was carried out as described [29]. In brief, 50 ml of 2xTY-Amp-Glu medium were inoculated with 10–20μl bacterial suspension to yield an OD600 = 0.05–0.1, and grown at 37°C and 225 rpm until OD600 = 0.4–0.5 (~ 6 x 10^9^ Bacteria). 15 ml of this culture were infected with helper phage VCS-M13 in a ratio of at least 20:1 (phage: bacteria) for 30–40 min at 37°C in a water bath. Typically, 100 μl VCS-M13 (~ 10^12^ pfu/ml) per 15 ml culture were used. The infected bacteria were centrifuged at 3.300 x g for 10 min at RT, resuspended in 100 ml 2xTY-Amp-Kana and incubated in a shaker at 30°C overnight. 100 ml of overnight culture were transferred to 250 ml buckets and centrifuged at 3.300 x g for 10 min at 4°C. The phage supernatant was transferred to new tube and ¼ volume of 20% polyethylene glycol (PEG)/ 2.5% NaCl was added for at least 1hr on ice. The mixture was placed on ice for 40 min and then centrifuged for 15 min at 10.800 x g at 4°C. Pellets were resuspended in 120 ml Aqua bidest/ 20% PEG/ NaCl (i.e. 24 ml) for a second precipitation step. After centrifugation, the supernatant was discarded and the pellet was resuspended in 3ml PBS/ 10% glycerol. The solution was rotated for 20 min at 37°C and centrifuged at 13.000 rpm for 2 min to remove any residual bacterial cell debris. Finally, phage solutions were transferred to fresh microcentrifuge tubes in 0.8 ml portions, which were stored at -80°C or used for the next panning round. For each subsequent panning step, ~10^12^ amplified phage particles of the previous enrichment round were used. A total of six consecutive panning rounds was performed on BECs and LECs by repeating this selection procedure.

### Phagemid preparation, DNA insert and fingerprint analyses and sequencing

Individual bacterial clones containing plasmids or phage replicative forms were resuspended in 2xTY/ 15% glycerol in 2ml plastic vials at -70°C for long term storage. 5ml 2xTY-media containing the appropriate antibiotic were inoculated with single bacterial colonies and incubated at 37°C in a shaker overnight. 1.5 ml of the overnight culture was centrifuged for 2 min at 3.000 x g to harvest bacteria. Plasmid or phagemid DNA was extracted using the Wizard Plus SV Miniprep DNA Purification System (Promega) or Wizard SV 96 Plasmid DNA Purification System (Promega) according to manufacturer´s instructions. For large scale DNA purification (up to 500 μg), endotoxin-free transfection-grade plasmid DNA EndoFree Plasmid Maxi Kit (Qiagen) was used following the manufacturer´s handbook. Clone diversity was determined by fingerprinting with the frequent-cutting enzyme *BstN*I (NEB, New England Biolabs) according to manufacturer’s instructions. DNA fragments were resolved on agarose gels run in TAE buffer. High throughput sequencing of single phagemid DNA was performed at Eurofins MWG Operon using primer 5´-TACTACGCAGACTCCGTGAAG-3´. Inserts were translated into correct amino acid sequences and phagemid clones were sorted according to frequency of respective CDR3 sequences. Homology trees of the CDR3 regions of variable heavy (VH) and light (VL) sequences were generated using clc main workbench (www.clcbio.com).

### Phage or scFv antibody preparation for ELISA screening

The versatile TG1 semisuppressor strain can be used for both, production of phage particles bearing pIII-fused scFvs by superinfection with helper phage and amber-suppression, or production of soluble scFvs by incomplete suppression of the amber stop codon [31]. Supernatants from clones of the 5^th^ and 6^th^ rounds of selection were screened by ELISA in phage and scFv format. Individual phage-infected TG1 colonies were grown in 96 deep-well plates (Nunc) in 150 μl 2xTY/ ampicillin/ 0.1% glucose. After 3 hrs, phage production was induced by addition of 25 μl 2xTY/ ampicillin/ 0.1% glucose, containing 1x10^9^ pfu helper phage VCS-M13. After infection for 30 min at 37°C, bacteria were pelleted and supernatants discarded. Infected bacteria were resuspended in 200 μl 2xTY/ ampicillin/ kanamycin and were grown overnight at 30°C. Bacteria were spun down at 1.800 x g for 10 min at 4°C, and 100 μl of the phage-containing supernatants were used for subsequent ELISA or FACS analyses. For scFv production, single colonies of phage-infected TG1 were grown in 5 ml 2xTY/ ampicillin/ 0.1% glucose, and scFv protein production was induced with 1mM IPTG for 16–24 hrs at 30°C. Bacteria were pelleted at 1.800 x g for 10 min and supernatants were immediately used in whole-cell ELISA. scFv antibodies were purified from bacterial culture supernatants by affinity chromatography using Protein AG UltraLink Resin (Pierce) according to manufacturer´s recommendations. The final antibody concentrations were either evaluated from OD_280nm_ (Nanodrop), by SDS-PAGE followed by coomassie staining, or by densitometric evaluation of Western blots probed with anti-His tag antibody.

### Phage amount and whole cell ELISA

For phage amount ELISA, wells of flat bottom Nunc-Immuno 96 MicroWell MaxiSorp Plates (Nunc; 436110) were coated with 500 ng (in 100μl) rabbit anti-fd bacteriophage antibody (Sigma; B7786) diluted in carbonate buffer (15mM carbonate, 35mM biscarbonate)/ pH9.6 or PBS/ pH7.4 overnight at 4°C. On the next day, wells were washed twice with 200μl TBST and blocked with 200 μl 5% MTBS for 30 min at RT. Screening of specific binding to cell surface antigens was performed by whole cell ELISA with phage antibodies or scFv fragments. BECs, LECs and additional cell lines were grown in 96-well plates to confluency. Cells were washed twice with PBS and fixed with PBS/ 1% PFA for 20 min at 4°C. Wells were washed 3 times with 200μl PBS and blocked with 200μl 2% MPBS for 30 min at RT. 100 μl of phage or scFv containing supernatants were added to wells in triplicates and incubated for 2 hrs at room temperature. Wells were washed 3 times with 200 μl TBST, and 100 μl of HRP conjugated anti-M13 monoclonal antibody (Amersham; 27-9421-01) diluted 1:5000 in 0.5% MTBS was added per well. After incubation for 30 min at RT wells were washed three times with 200 μl PBS and incubated with 1:1000 diluted HRP-conjugated anti-M13 polyclonal antibody (Amersham Pharmacia) in 2% MTBS. Final washing was performed 3 times with 200 μl TBST and once with 200 μl TBS and inverted plates were blotted on a clean paper towel to remove any remaining washing buffer. The assay was developed with 3,3′,5,5′-Tetramethylbenzidine (TMB) Liquid Substrate System for ELISA (Sigma-Aldrich). Color reaction was stopped by adding of TMB stop reagent (Sigma-Aldrich), and color absorbance was immediately read at 450 nm in an ELISA plate reader (Bio-Tek).

### FACS analysis with phage antibodies

Binding of phage particles or phage-displayed scFvs to HDMECs, BECs or LECs was determined by flow cytometry. Cells were plated at a cell density of 1 x 10^5^ cells/ cm^2^ in small culture flasks. After 72 hours, cells were gently detached from the culture dish by EDTA, washed twice with PBS and kept on ice. For each FACS analysis sample, aliquots of 5x10^5^ cells were blocked with 2% MPBS/ 0.05% NaN_3_ for 30 min on ice, and approximately 5 x 10^12^ phage particles were blocked with 2% MPBS for 1 h at room temperature. For each determination, cells were resuspended in 100 μl FACS buffer (1% FCS, 0.01M EDTA/ PBS) mixed with blocked phage particles in micronic tubes, and incubated on ice for 1 hr. Cells were washed twice with 500 μl 2% MPBS, incubated with rabbit anti-M13 phage antibody (S1 Table) diluted 1:500 in 2% MPBS for 30 min on ice, washed twice with 2% MPBS and incubated with FITC-labeled goat anti-rabbit antibody diluted 1:1000 in 2% MPBS for 1 hr on ice. In case of scFv staining, a directly Alexa Fluor 488 conjugated anti-His-tag antibody, diluted 1:200 in FACS buffer, was used. Samples were washed twice with PBS, resuspended in 500 μl PBS and analyzed on a FACScalibur device (Becton Dickinson) using software FCS Express 2.0.

### Immunoprecipitation

BECs (5x10^6^) were washed twice with ice cold PBS and lysed with 500μl lysis buffer (TBS pH7.4/ 1% NP40/ complete protease inhibitor cocktail) for 10 min on ice. Cell lysates were centrifuged for 5 min, 14.000 rpm at 4°C and cleared supernatant was transferred into a new tube. For protein deglycosylation, 30 μl of BEC lysate containing 30 μg protein (1 μg / μl) was incubated with 750 units of PNGase F (NEB, New England Biolabs) for 3 hrs at 37°C. For immunoprecipitations, one half of the lysate was incubated with 5 μg scFv, and the other without (negative control) at 4°C overnight under rotation. To circumvent failure of specific antigen enrichment (internal communication), we chose Protein A/G UltraLink Resin (Pierce) with high ability to capture VH3 segment containing scFvs [34]. Immune complexes were captured with 25 μl of Protein A/G Resin and incubated for 1hr at 4°C under rotation. Samples were centrifuged for 3 min, 2.000 rpm at 4°C and the pellets were resuspended four times with lysis buffer, each time performing transfer to a fresh tube. The pelleted resins were incubated at 95°C for 5 min with 2 x SDS sample buffer and either used for subsequent SDS-PAGE and Western blot analysis, or were sent to mass spectrometry facility for further processing.

### SDS-PAGE and Western Blotting analyses

Proteins were denatured at 95°C for 5 min, and 20 μl of protein lysates were separated per slot on a 12% bisacrylamide/ acrylamide gel. Proteins were transferred overnight to nitrocellulose membranes (BA83, poresize 0.2 μm, Whatman Protran). Membranes was dried between 20 min and overnight and blocked with TBST/ 5% BSA. Membranes were washed three times for 10 min in TBST at RT and incubated with indicated antibodies in TBST/ 5%BSA for 1 hr at RT, or overnight at 4°C (depending on the applied antibody) (S1 Table). After incubation with the first antibody, membranes were washed three times for 10 min in TBST and incubated with HRP-conjugated secondary antibodies for 1 hr. Membranes were washed five times for 10 min in TBST and chemoluminescent signals were developed with ECL plus (GE) reagent. Lumi-Imager F1 (Boehringer, Mannheim) system was used to detect chemoluminescent signals, exposure times varying between 5 sec and 10 min, depending on the signal intensity.

### NanoLC ESI MSMS

Samples were subjected to LC MS/MS analysis at the APAF (www.proteome.org.au) according to standard sample preparation for mass spectrometry. Briefly, proteins were released from the resin by incubation in 300 μl of 0.05% SDS/ 0.5M triethylammonium bicarbonate (TEAB) at 95°C for 15 min. The resin was centrifuged and the supernatant was collected. Tris (2-carboxyethyl) phosphine hydrochloride (TCEP) and methyl methane thiosulfonate (MMTS) were added to reduce and alkylate the proteins. Subsequent trypsin digestion was performed overnight at 37°C. Cation-exchange Macro-Prep High S Support columns (Bio-Rad) were used to clean the samples. Digested peptides were separated by nano-LC using the Tempo nanoLC system (Applied Biosystems, CA, USA). The sample (~1/3 of the original amount) was preconcentrated on a peptide trap (Michrome peptide Captrap) and desalted with H_2_O/ 2% CH_3_CN/ 0.1% formic acid at 8 μl / min. The peptide trap was then connected with the analytical column containing C18 RP silica (SGE ProteCol C18, 300A, 3μm, 150μm x 10cm). Peptides were eluted from the column using a linear solvent gradient of H_2_O:CH_3_CN (0.1% formic acid) from 90:10 to 0:100% (v/v) at 500 nl / min over a period of 50 min. The LC eluent was subjected to positive ion nanoflow electrospray analysis on an Applied Biosystems QSTAR Elite mass spectrometer. The QSTAR was operated in an information dependant acquisition mode (IDA). In the IDA mode, a TOFMS survey scan was acquired (m/z 400–1600, 0.5 sec), with the three largest multiply charged ions (counts >25) in the survey scan sequentially subjected to MS/MS analysis. MS/MS spectra were acquired in dynamic exit mode with a maximum accumulation time of 2 seconds. The LC-MS/MS data were analyzed using Mascot Server (http://www.matrixscience.com) to search peptide alignments to ‘Homo sapiens’ entries in the NCBInr and SwissProt protein databases.

### Recombinant pFUSE antibody engineering, eukaryotic cell expression and purification

scFv-mFc fusion antibodies were generated by PCR-amplification of a 732 bp portion of scFvs using 5’-TACGGCAGCCGCTGGATTGTTATTACTC-3’ as forward, and 5’-TTATTAGATCT-CGCGCCTAGGACGGTCA-3’ as reverse primers. After restriction digest with *Nco*I and *Bgl*II, scFv fragments were inserted in correct reading frame between the IL2 secretion signal and the murine Fc sequence of pFUSE-mIgG2B-Fc2 expression vector (Invivogen) (S5C Fig). Ligation products were transfected into competent TG1 E.coli, and plasmids were prepared from single clones (Qiagen). One day before transfection, cultured HEK293 cells were split and seeded at a density of 3x10^6^ cells per 10 cm cell culture dish in 10 ml antibiotic free medium (DMEM/ 10% FCS). Transfection of HEK293 cells was performed using Lipofectamine 2000 (Invitrogen) according to the manufacturer´s instructions. Cell culture supernatants were collected 72 hrs after transfection and antibodies were purified with proteinAG UltraLink Resin (Pierce), yielding scFv-Fc antibody concentrations of 0.2–2 mg/ml. Supernatants containing Fc alone (from vector alone transfectants) was similarly processed and used as negative control.

### Recombinant CD146 ELISA

Recombinant human CD146 comprising the extracellular Met^1^-Gly^559^ portion of the protein precursor (NP_006491.2) fused to a C-terminal poly-Histidine tag was purchased commercially (Sino Biological Inc., Nr. 10115-H08H). ELISA plates were coated with CD146 or BSA as control antigen at concentrations of 100, 10 and 1 ng / ml in triplicates overnight at 4°C and were blocked with TBST / 5% BSA for 2hrs on the following day. Commercially available antibodies, scFv fragments and scFv-Fc fusion antibodies were added serially diluted to respective wells and incubated at RT overnight, followed by HRP-conjugated detection antibodies. ELISA plates were washed and developed as described above. In Western blotting, cell lysates were electrophoresed in 12% SDS-PAGE and transferred to nitrocellulose. Blots were probed with scFv, scFv-Fc and commercially available antibodies (S1 Table).

### Scratch wounding assay

For migration assay, confluent BEC monolayers grown in 24-well plates (n = 3) were starved overnight. Thereafter, an artificial wound was created using a 200 μl pipette tip. Non-adherent cells were washed away and BECs were treated with or without 10 μg/ml of purified scFv-Fc B6-11 or Fc fragment only in EBM-2 MV / 0.5% FCS for 48 hrs. Wound closure was monitored with an inverted live cell microscope (AxioVert 200M, Zeiss) in an incubation chamber by taking pictures of the same section (2 sections per scratch) every hour over the whole time period. Wound area was measured using AxioVision 4.7 and calculated as amount of wound closure (in %) from timepoint t = 0. The experiment was performed twice.

### Statistical data analysis

Statistical analyses were performed using Graph Pad Prism Software. Gap closure was analysed by one-way Anova followed by pairwise comparison to the control groups using paired t-test considering a *P*-value ≤ 0.05 as significant.

## Results

### Establishment of scFv phage selections on primary BECs and LECs

**Fig 1:**
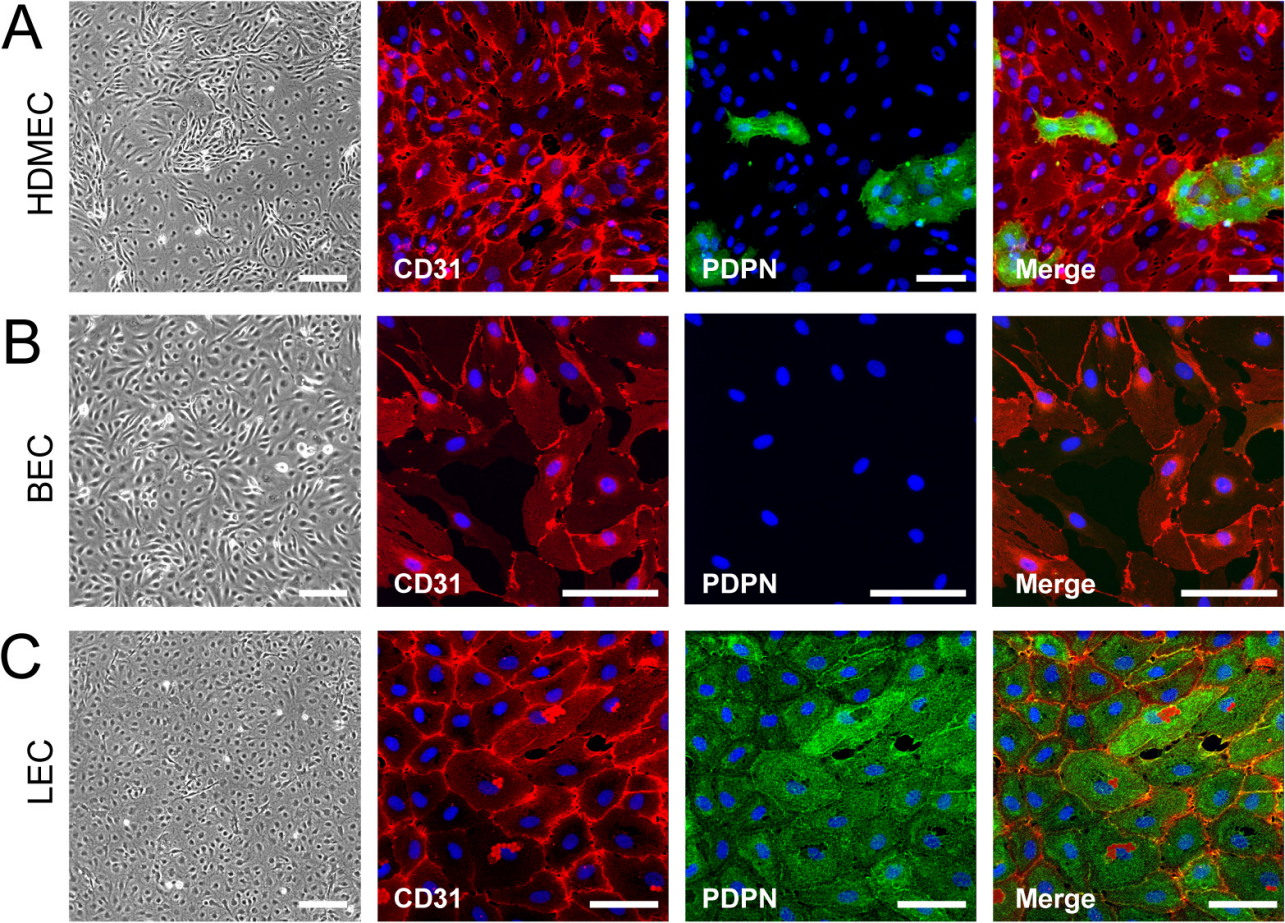
Purity control of HDMEC, BEC and LEC cultures used for *in vitro* cell panning experiments. Light microscopy and double-immunofluorescence stainings of HDMECs that were separated into BECs and LECs by sorting with magnetic beads coated with rabbit anti-podoplanin (PDPN) antibody. A: HDMECs contain subcultures of BECs and LECs. B: BECs are CD31+ / PDPN-. C: LECs are CD31+ / PDPN+. Nuclei were counterstained with DAPI. Size bars: 50μm.

Primary human BECs and LECs were retrieved from initially mixed HDMEC cultures (S1 Fig). Successful separation of BECs and LECs was tightly controlled by immunofluorescence staining of endothelial marker molecules CD31 and podoplanin (PDPN) (Fig 1) and by established PCR control (not shown), as described previously [8]. Cells were used only up to six passages, as long-term culturing leads to marker destabilization [8]. The semi-synthetic antibody phage library ETH-2 [29] comprising a variety of 5 x 10^8^ phage clones was used for panning on BECs and LECs. Selection parameters were determined empirically: First, selections were performed with cell suspensions, as panning on cells grown in monolayers predominantly yielded plastic and serum protein binders [16]. Second, as epitope internalization might occur at higher temperatures, we panned at 4°C. Third, as cell-bound phages are hardly recovered by pH-shift [16,35] (own unpublished results), phages were not eluted chemically from the cells, but cell-bound phages were directly added to logarithmically growing TG1 *E*. *coli*. In parallel, selections were performed with identical numbers of VCS-M13 wildtype phage (S2 Fig) to determine background-binding of phage particles carrying no antibodies. Trypan blue cell-staining showed that the cells had not been lysed during phage incubation and washing procedure (not shown), indicating a high chance to select scFvs against cell surface proteins.

### Characterization and binding specificities of polyclonal phage antibody eluates

**Fig 2:**
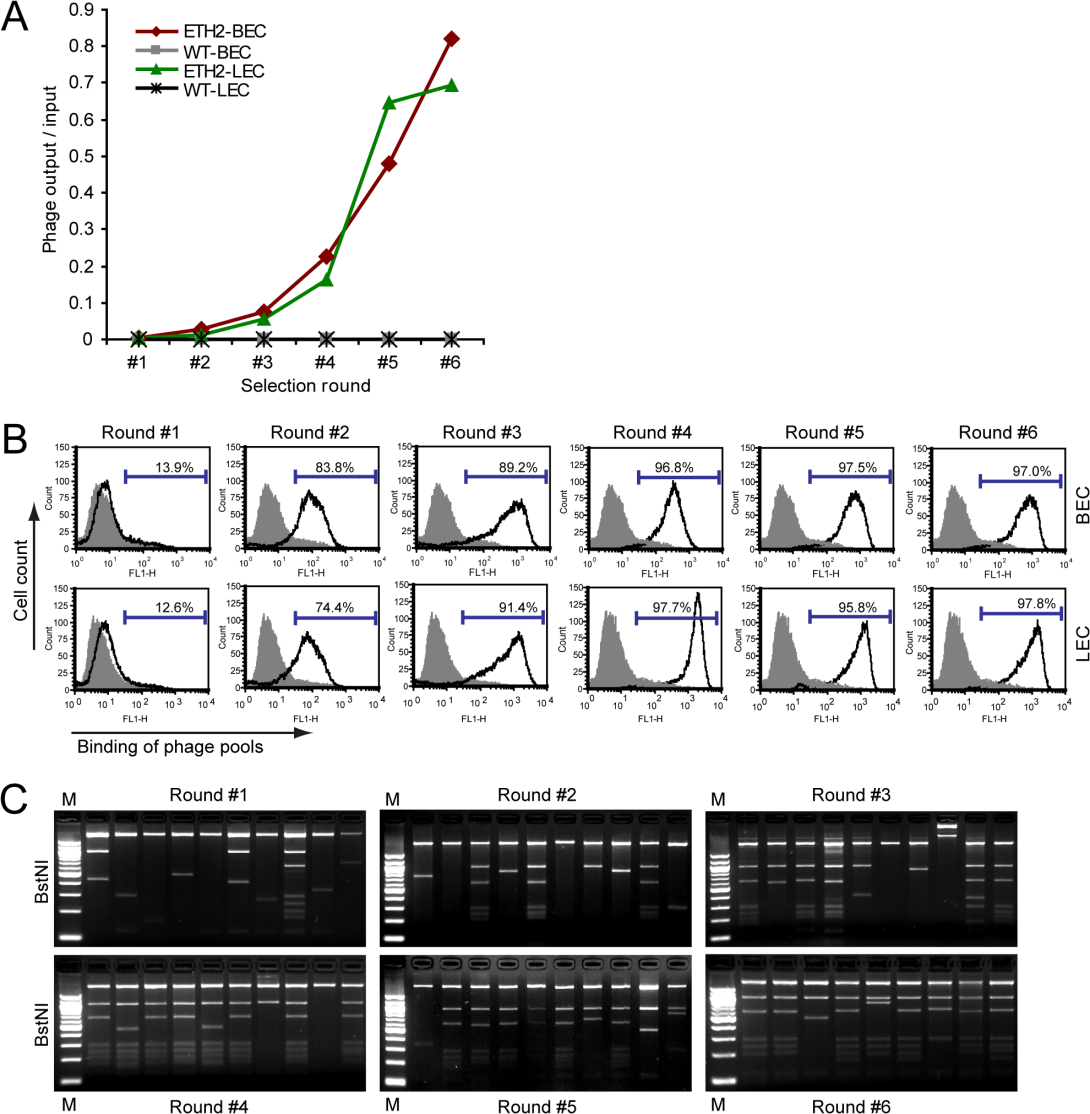
Enrichment of phage antibodies that bind to BECs and LECs with successive biopanning rounds. A: Numbers of recovered phage particles after each panning round on BECs and LECs. For each selection round, the phage output/input titer ratios are given on the y-axis, showing an increase on BECs (red line) and LECs (green line) when adding polyclonal ETH-2 phage. In contrast, control wild-type VCS-M13 (grey and black lines) phage did not shift significantly on either cell population. B: Enrichment of BEC- and LEC-binding phage antibodies as seen by FACS-analysis of phage antibody pools. Shown are representative histograms of BECs and LECs stained with phage antibodies after respective panning rounds (black line) and WT phage as negative control (grey-filled graphs). The number of cells (counts: Y-axis) is given as function of the fluorescence intensity of phage antibody staining of the cells (FL1-H: X-axis). Percentage of BECs and LECs shifted by phage binding is depicted in the graphics. In all experiments, cells were incubated with phage antibody pools, and cell-binding was detected by anti-M13 antibody and FITC-conjugated anti-rabbit antibody. Grey-filled graphs show WT phage binding as negative control. C: *BstN*I DNA fingerprint analysis of single scFv clones. Representative gel images of respective panning rounds (#1 - #6) performed on BECs and LECs are shown. Over the course of panning rounds, the number of intact clones is increasing. Phagemid DNA isolated from single clones was digested with *BstN*I and analyzed in agarose gel electrophoresis. M: 1 kbp molecular weight marker.

Established enrichment steps were performed six times on separate BEC and LEC suspensions. Cell-specific phage antibody recoveries and enrichment factors were calculated for each panning round (Table 1). Phage recoveries and enrichment factors of ETH-2 selections showed an exponential increase on BECs and LECs, while they remained at constant low levels when applying wildtype phages (Table 1, Fig 2A). Phage antibody enrichment was not enhanced by increased cell numbers or by performing more than six selection rounds (not shown). Concomitantly, a FACS analysis of polyclonal phage pools confirmed binding of cell-enriched phage antibodies (Fig 2B, black-lined graphs), but not of VCS-M13 wildtype phages (Fig 2B, grey-filled graphs), to BECs (upper row) and LECs (lower row). Next, phagemid DNA derived from randomly picked single clones of all selection rounds was analyzed by *BstN*I-digest. While the restriction pattern differed widely during panning rounds #1 - #3 and restriction-resistant clones were detected (Fig 2C, upper row), rounds #4 - #6 mainly consisted of intact scFv clones (Fig 2C, lower row). These data showed that six selection rounds had resulted in enrichment of phage antibodies specifically binding to BECs and LECs, relative to the original antibody library complexity.

### scFv antibody sequencing and bioinformatic analysis

Phagemid DNA of 994 individual single antibodies derived from selection rounds #5 and #6 was sequenced, and corresponding amino acid sequences were determined, yielding 557 intact antibody clones (i.e. 56%). From these, 166 diverse antibodies were derived, as exemplified by their CDR3 region sequences (S2 Table). A more detailed sequence statistics revealed that the majority of the clones was unique (Table 2) and contained CDR3 insert lengths of 6 amino acids (S3 Table). Further, a homology alignment of VH and VL CDR3 inserts indicated a broad diversity of the sequences, excluding biased clone enrichment (S3 Fig). In conclusion, the scFv sequence analysis indicated a highly diverse, unbiased clone abundance in phage selection rounds #5 and #6, out of which we isolated 166 diverese phage antibodies.

**Table 2:** Frequency of diverse scFv antibody sequences among 557 intact clones.

scFv sequences ^a^	scFv sequence frequency ^b^	scFv sequence count ^c^	scFv sequence occurrence [%] ^d^
1	182	182	32.7
1	29	29	5.2
1	27	27	4.8
1	17	17	3.1
1	12	12	2.2
1	8	8	1.4
1	5	5	0.9
2	13	26	4.7
2	9	18	3.2
7	4	28	5.0
9	3	27	4.8
21	2	42	7.5
115	1	115	20.6
**166**		**557**	**100%**

Out of 994 sequenced phage clones, 557 intact scFv sequences were derived, among which 166 diverse scFv sequences were identified (see S2 Table).

^a^ Frequency of unique scFv antibody sequences

^b^ Number of different scFv sequences

^c^ Number of scFv sequences with respective sequence diversity

^d^ Sequence occurrence was calculated as percentage of sequence count.

### Single phage antibodies specifically bind to BECs and LECs

**Fig 3:**
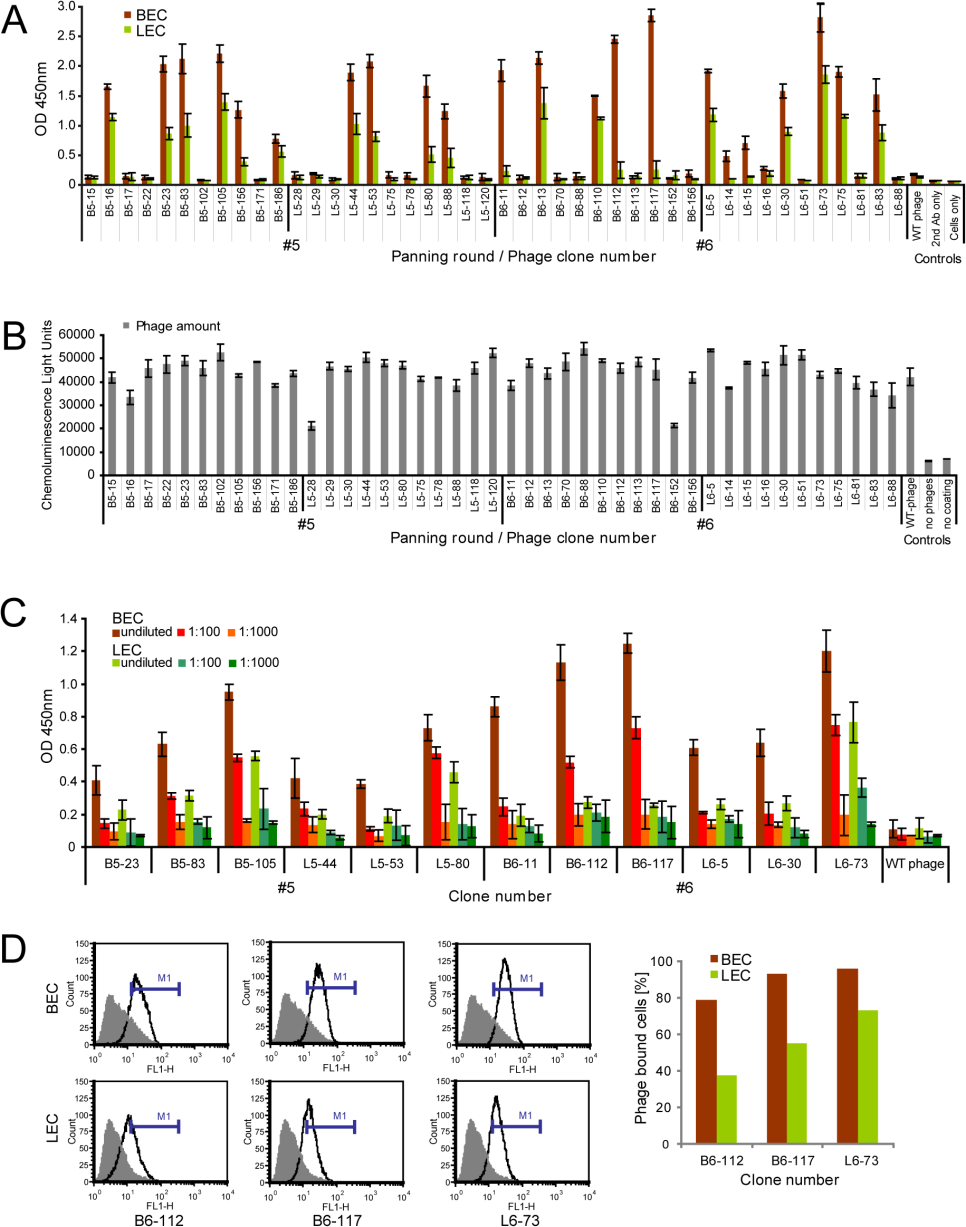
Binding assessment of single phage antibodies to BECs and LECs in cell ELISA and FACs. A: Representative whole cell ELISA of phage antibodies prepared from 166 unique scFv clones derived from panning rounds #5 and #6 on BECs (red) and LECs (green). Values represent mean ± SD (n = 3). Negative controls were: WT-phage, 2xTY medium without phage, 2^nd^ antibody only. Phage antibody binding was visualized via peroxidase-conjugated anti-M13 phage antibody. OD value represents absorbance at 450nm. B: Phage amount ELISA of single phage antibodies. To allow for normalization of cell ELISA signal intensities, phage antibody concentrations were determined in a second ELISA format, where anti-fd bacteriophage antibody (S1 Table) was coated, and phage containing supernatants were added. Bound phage particles were detected by peroxidase-conjugated anti-M13 phage antibody (S1 Table). C: Effect of decreasing numbers of phage antibody concentrations on binding to BECs and LECs by cell-ELISA. Phage antibody dilution series in cell ELISA assessing specific binding to BECs and LECs. Control: same dilutions of WT phage. D: Binding of three selected phage antibodies to BECs and LECs analyzed by flow cytometry. Shown are histograms of BECs and LECs stained with the three phage antibodies (black lines). Grey-filled graphs show VCS-M13 wildtype phage binding as negative control. Cell binding was detected by sequential incubation with anti-M13 and FITC-conjugated anti-rabbit antibodies. The percentage of BECs and LECs bound to phages was evaluated by setting a marker (M1) indicated by the blue bar and is depicted in the graph.

All 166 intact phage antibodies were produced in large scale and screened for binding to BECs and LECs by whole-cell ELISA (Fig 3A). Presence of identical cell amounts in different wells was controlled by light microscopy, using confluent monolayers (Fig 1). In parallel, supernatants of the same antibodies were applied in a phage amount ELISA to normalize the phage antibody binding signals on cells (Fig 3B). Overall, 86 clones (53%) reacted with ECs by binding at least 3-fold over the background signal produced by WT phages. The other clones reacted weaker towards the cells, probably due to off-rates higher than the threshold set by the screening ELISA, or by recognizing underrepresented antigens. Importantly, though with different affinities, nearly all clones bound to both cell lines, BECs and LECs (Fig 3A), indicating a strong overlap of surface markers between these two lineages. Although the enrichment curve and FACS analysis had shown a clear increase of LEC-binders (Fig 2A and 2B), no exclusive LEC-specific antibodies were identified, and also depletion selection and competitive elution were not successful to derive LEC-specific clones (not shown). Twelve phage antibodies showing eminent reactivity in cell ELISA were picked for detailed characterization. Cell binding specificity was assessed by applying dilution series of selected phage antibodies (Fig 3C), and FACS analysis of the three strongest binders reconfirmed specific EC-reactivity (Fig 3D). Hence, the selection had yielded a panel of high-affinity BEC- and LEC-binding phage antibodies.

### Soluble expression of scFvs and phage-independent binding to BECs and LECs

**Fig 4:**
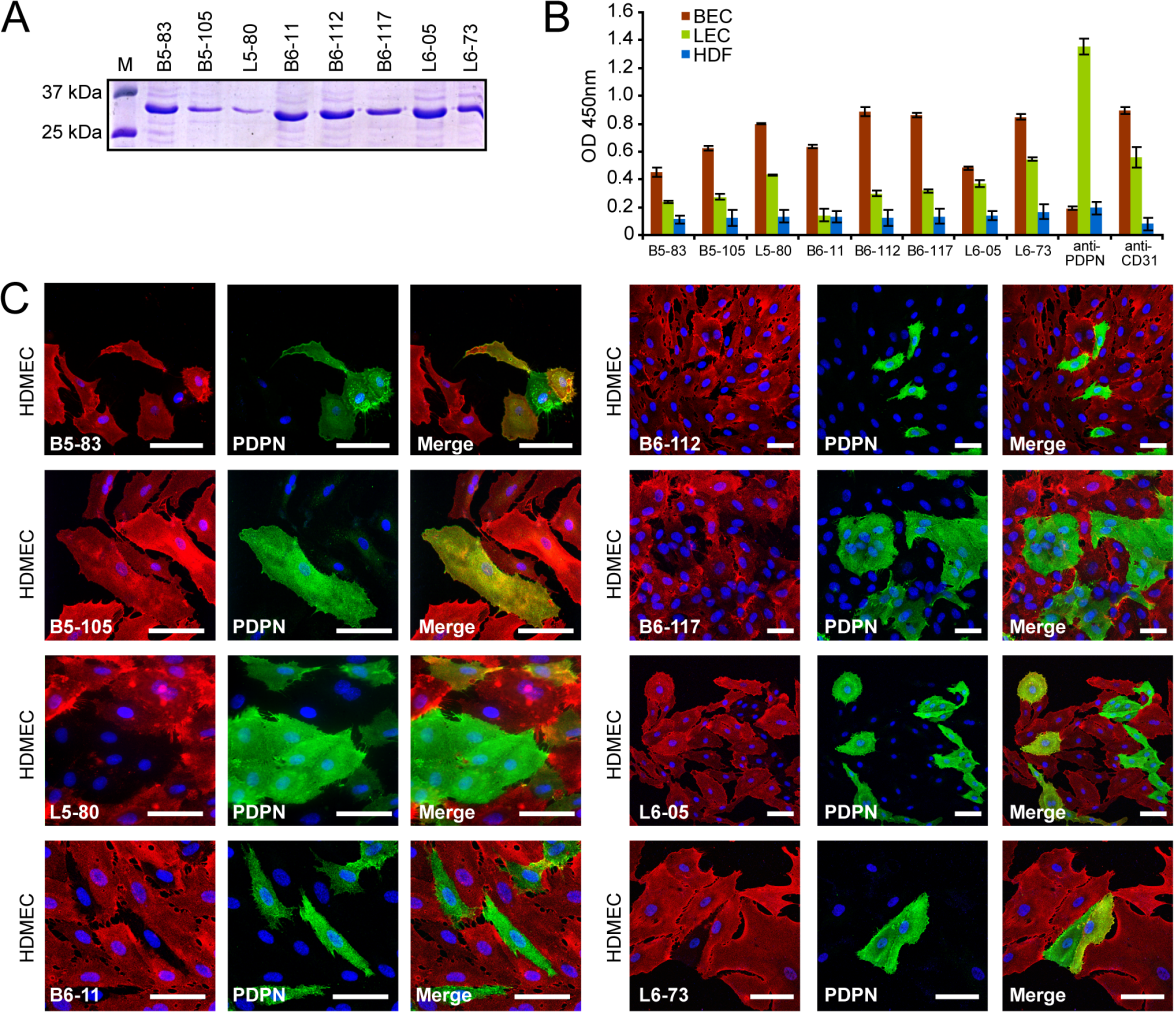
scFv antibody production and binding to BECs and LECs in cell ELISA and immunofluorescence. A: SDS-PAGE analysis of scFv antibody expression. Eight single scFv clones were grown in *E*. *coli* TG1 and scFv production was induced by IPTG. scFvs were purified from culture supernatants by using protein A sepharose and elution with glycine buffer at pH 2.2. Samples were captured and adjusted to PBS on Zeba spin desalting columns (Thermo Scientific). Proteins were separated on a 10% SDS-polyacrylamide gel and stained with Coomassie blue for molecular weight detection. Typical purification results of eight clones are shown with Mr in the range of 30 kDa. B: Whole cell ELISA with soluble scFv antibodies prepared from eight phage clone cultures. BECs, LECs and human dermal fibroblasts (HDF) cultivated in 96-wells were incubated with scFv fragments purified from bacterial culture supernatants. Comparison with anti-podoplanin and anti-CD31 antibodies as positive control. C: scFv antibodies show different labeling patterns on HDMECs in immunofluorescence. Representative double immunofluorescence images showing staining of eight different monoclonal scFv antibody clones on HDMECs. As seen from co-immunofluorescent staining against podoplanin (green), B5-83, B5-105, L5-80, L6-05 and L6-73 showed overlapping staining for BECs and LECs, while B6-11, B6-112 and B6-117 revealed specific BEC staining. Nuclei were counterstained with DAPI. Size bars: 50μm.

According to high binding affinity and antibody sequence abundance, eight antibodies were chosen for further analysis. Soluble scFv fragments were produced in *E*. *coli* non-suppressor strain (Fig 4A) and purified from culture supernatants, which resulted in 0.2–0.5 mg / ml culture soluble scFv antibody. Binding of this scFv antibody panel to BECs, LECs and human dermal fibroblasts (HDFs, see S1 Materials and Methods) as control cell line was re-examined in cell ELISA. The background was low, and when diluted 1:10, signals were in the range of commercial anti-PDPN and anti-CD31 antibodies (Fig 4B). All scFvs retained binding capacity towards BECs and LECs, with similar reactivities as the corresponding phage antibodies (Fig 3C). Immunoprobing of BEC and LEC lysates with scFv antibodies under reducing and non-reducing condition did not yield specific signals (not shown), indicating that binding of scFvs required native antigen. When tested in immunofluorescence on HDMECs, all eight scFv antibodies revealed a membrane-associated staining pattern (Fig 4C), as would be expected from the whole cell-selection procedure. A more detailed inspection by co-staining against podoplanin revealed that one antibody (B5-105) reacted equally to both, BECs and LECs, four antibodies stained BECs in combination with subpopulations of LECs (B5-83, L5-80, L6-05, L6-73), while three scFv antibodies showed selective BEC-labeling (B6-11, B6-112, B6-117). In accordance with previous cell ELISA screenings (Fig 3A), there was no LEC-specific staining. These data proved that soluble, phage-derived scFv antibodies retained binding capacity to BECs and LECs, out of which we identified three BEC-specific antibodies.

### Antigen retrieval for clone scFv B6-11 identifies CD146/MCAM

**Fig 5:**
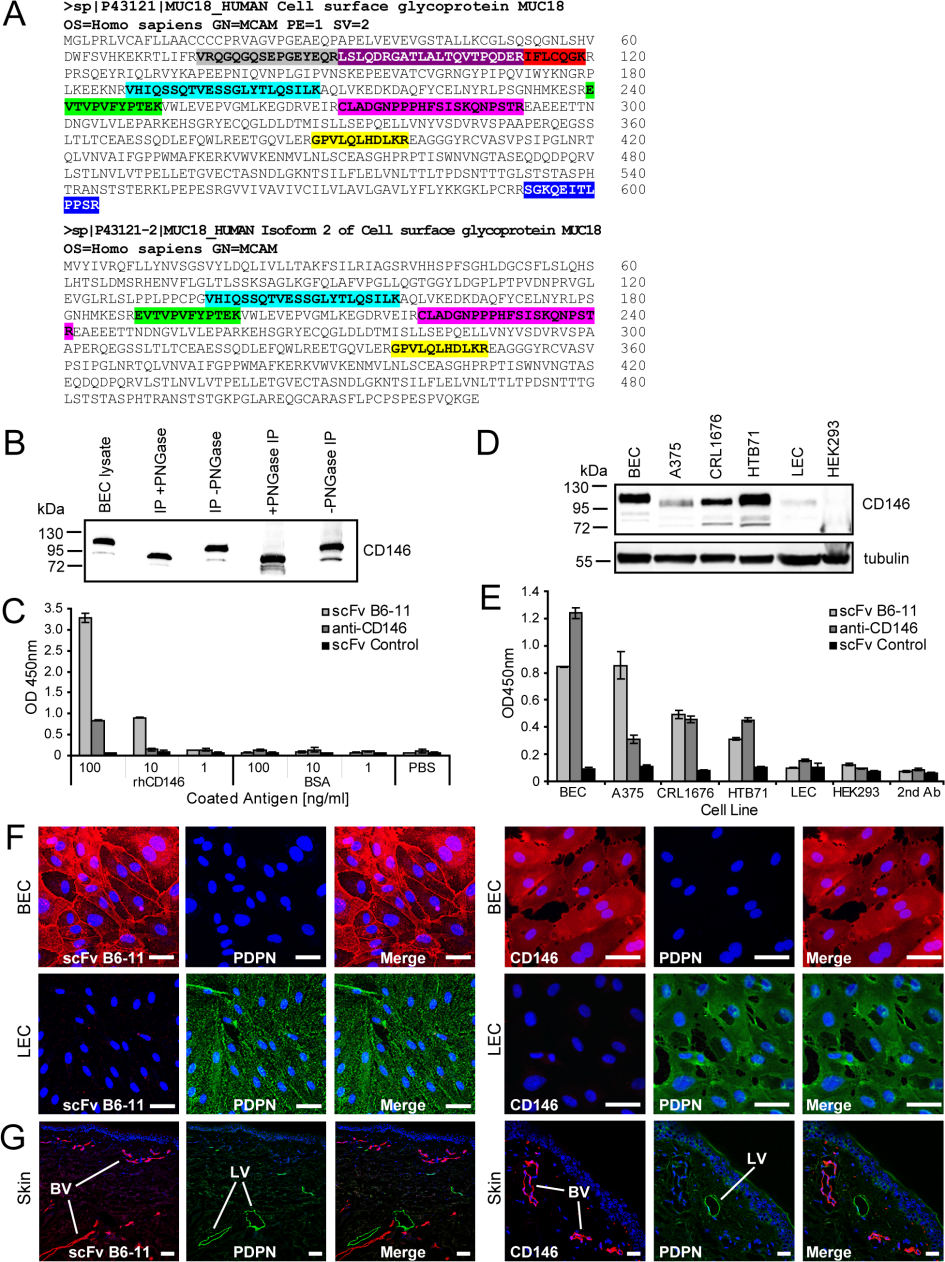
LC-MS/MS identification of CD146 binding to scFv B6-11, and antigen confirmation by immunoprecipitation and ELISA. A: Alignment of LC-MS/MS identified peptides (S4 Fig) with the sequence of MCAM/CD146MUC18. The eluates from scFv B6-11 immunoprecipitation were subjected to trypsin digestion (see Materials and Methods section) and subsequently analyzed by LC-MS/MS. B: Immunoprecipitation of CD146 by soluble scFv B6-11 from BEC lysates. Immune complexes were tested by Western blot in reducing conditions using commercial anti-CD146 antibody. Lane 1: input BEC lysate, lanes 2–5: immunoprecipitates with scFv B6-11, lanes 2 and 4: under addition of PNGase F. Treatment of BEC lysates with PNGase prior or after addition of scFv B6-11 had no influence on co-immunoprecipitation capacity of scFv B6-11, showing that scFv B6-11 binding to CD146 is glycosylation-independent. C: scFv B6-11 binds to immobilized extracellular domain of recombinant human CD146 in ELISA. An irrelevant antigen (BSA), a non-binding scFv and uncoated wells served as controls. scFv binding was detected with peroxidase-conjugated anti-His tag antibody (S1 Table), and absorbance was measured at 450nm. Mean and standard deviation of triplicate experiments are given. D: CD146 expression of different cell lines as shown by immunoprobing with anti-CD146 antibody. CD146 is expressed in BECs, in A375, CRL1676, HTB71 melanoma cells, but not in primary LECs and HEK293 cells. The same blot was probed with anti-tubulin antibody for control of equal protein loads. E: scFv B6-11 stains cell lines expressing CD146 with similar intensity as commercial anti-CD146 antibody in ELISA. Negative controls were a non-binding scFv and 2^nd^ antibody only. F: Similar to commercial anti-CD146 antibody, scFv B6-11 stains BECs (upper lane, red) but not LECs (lower lane, green) in immunofluorescence. Size bars: 50μm. G: scFv B6-11 stains blood, but not lymphatic vessels in human skin. Double immunofluorescence staining of skin sample with Cy3-labeled scFv-B6-11 or anti-CD146 (red) and anti-PDPN (green) antibodies. Blood (BV) and lymphatic (LV) vessels are indicated by lines. Nuclei were counterstained with DAPI. Size bars: 50μm.

Showing highest specificity for BECs versus LECs (Fig 4B and 4C), we aimed to identify the antigen bound by scFv B6-11 antibody. Therefore, BEC lysates were used for immunoprecipitations with soluble scFv B6-11. Although no obvious band was detected in SDS-PAGE analysis of immune complexes (not shown), immunoprecipitates were subjected to trypsin digest and subsequent LC-MS/MS analysis. MS/MS spectra of the sample were searched in the Mascot database, which identified hits in the scFv B6-11 list that were clearly distinct from controls (S4 Fig). Nine peptides of the main hit showed exact identity with MUC18 surface glycoprotein (NP_006491), also known as CD146 / MCAM (Fig 5A). CD146 is a cell adhesion molecule belonging to the Ig super-family proteins [36]. Its mucin-type 71 kDa protein core is highly glycosylated, which brings the total molecular mass to 113–130 kDa, depending on the cell type. To test whether scFv B6-11 was specific for CD146, we followed several strategies: Immune complexes captured from BEC lysates with scFv B6-11 were probed with commercial anti-CD146 antibody, revealing a band at approximately 120 kDa (Fig 5B) that corresponds to the predicted molecular weight of CD146. Further, treatment of BEC lysates before and after addition of scFv B6-11 with PNGase F, which removes oligosaccharides from N-linked glycoproteins, led to a down-shift of the detected band to 95 kDa (Fig 5B), indicating that scFv B6-11 bound to CD146 independent of post-translational glycosyl-modification. As scFv B6-11 did not detect CD146 in immunoblotting (not shown), direct binding of scFv B6-11 was analyzed using an immobilized recombinant protein representing the Met^1^-Gly^559^ portion of human CD146 (see Materials and Methods) in ELISA. scFv B6-11 as well as the commercial anti-CD146 antibody, but not control scFv B6-117, bound concentration-dependent this domain compared to control antigen (Fig 5C). These data suggested that scFv B6-11 bound to CD146 in native condition.

### scFv B6-11 labels native CD146/MCAM on BECs and blood vessels

CD146 was initially identified in melanoma and later also on endothelial cells [37,38]. Screening a panel of cell lines with commercial anti-CD146 antibody in immunoblotting showed that CD146 was highly expressed in BECs and in melanoma cell lines A375, CRL1676 and HTB71, but not in LECs and HEK293 cells (Fig 5D). Application of scFv B6-11 besides the commercial anti-CD146 antibody on these cell lines in ELISA revealed comparable reactivity (Fig 5E), suggesting that both antibodies recognized the same antigen. Further, when applied in immunofluorescent stainings, scFv B6-11 separately labeled BECs, but not LECs (Fig 5F, left panel), consistently with differential labeling of BECs versus LECs with anti-CD146 antibody (Fig 5F, right panel). Moreover, scFv B6-11 proved discriminatory labeling of blood versus lymphatic vessels in human skin cryosections (Fig 5G, left panel), similar to CD146 that did not colocalize with podoplanin-positive lymphatic capillaries (Fig 5G, right panel). These results indicated that phage derived scFv B6-11 recognized a conformational epitope of CD146 present on BECs and melanoma cells, and showed that B6-11 specifically labeled dermal blood versus lymphatic vasculature.

### scFv-FcB6-11 fusion antibody retains specificity for native CD146

**Fig 6:**
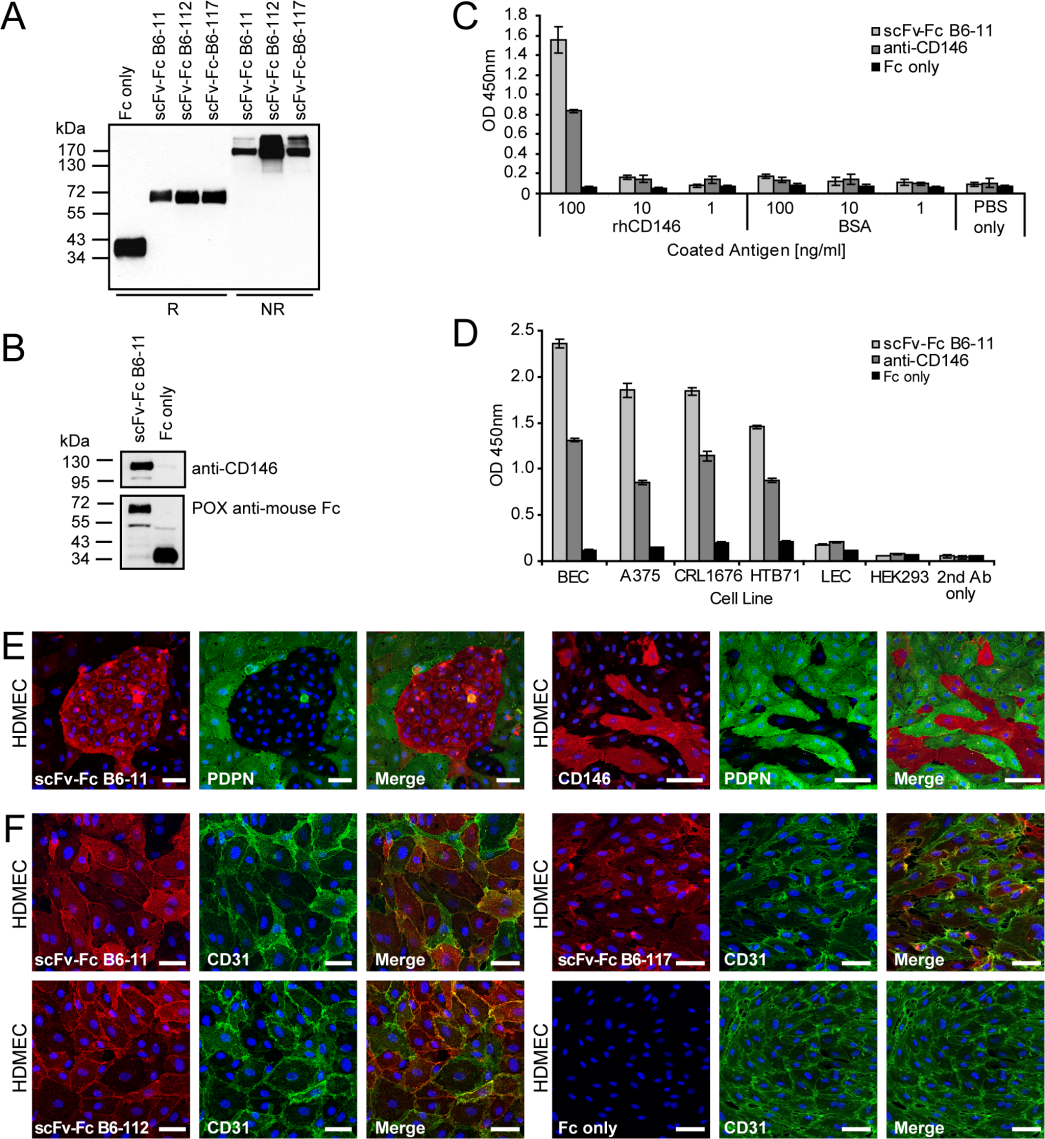
Production of scFv-Fc B6-11 fusion antibody and reconfirmation of binding to CD146. A: Western Blot analysis showing molecular size of scFv-Fc fusion antibodies B6-11, B6-112 and B6-117. The scFv-region of the phagemids was cloned into the pFUSE-mIgG2B-vector (see S5 Fig). Constructs were transfected into HEK293-cells. Cell culture supernatants were loaded on reducing (R) and non-reducing (NR) 10% SDS-PAGE, and nitrocellulose blots were probed with peroxidase-labeled anti-mouseFc antibodies. scFv-Fc fusion antibodies are secreted as approximate 140 kDa dimers, as seen under non-reducing condition (-DTT). Control: Fc only protein, produced from pFUSE vector without scFv insert. B: Immunoprecipitation with scFv-Fc B6-11 reconfirms CD146-binding. G1S1 lysates were incubated with scFv-Fc B6-11 and Fc only as control. Blots of immunoprecipitates were probed with anti-CD146 and anti-mouseFc antibodies. C: scFv-Fc B6-11 binds to recombinant CD146 in ELISA. scFv-Fc B6-11, commercial anti-CD146 antibody and Fc only were used on respective dilutions of recombinant human CD146 or BSA as control antigen coated on 96-well ELISA plates. Absorbance was measured at 450nm. D: scFv-Fc B6-11 fusion antibody binds to cells expressing CD146 in ELISA. Purified scFv-Fc B6-11 (light grey bars), commercial anti-CD146 antibody (dark grey bars) and Fc only (black bars) were added to monolayers of respective cell lines. Bound antibodies were detected using anti-Fc and HRP-conjugated anti-rabbit antibodies, and absorbance was measured at 450nm. Mean and standard deviation of triplicate experiments are given. E: On HDMECs, scFv-Fc B6-11 fusion antibody shows the same reactivity as a commercial anti-CD146 antibody. F: scFv-Fc fusion antibodies (red) reveal diverse membraneous labling patterns in co-immunofluorescent stainings with anti-CD31 antibody (green). Nuclei were counterstained with DAPI. Size bars: 50μm.

We next examined whether the three scFvs B6-11, B6-112 and B6-117 retained BEC-specificity as scFv-Fc fusion antibodies (S5A Fig) expressed in eukaryotic HEK-293 cells. By subcloning into the eukaryotic expression vector pFUSE mIgG2B-Fc2 [39] (S5B Fig), scFv regions (S5C Fig) were fused to a murine IgG Fc region. Immunoblotting of cell culture supernatants with anti-mouseFc-IgG antibody showed that scFv-Fc fusion antibodies migrated as monomers of approximately 80 kDa under reducing condition (R) (Fig 6A, lanes 2–4), while they behaved as dimers of 160 kDa under non-reducing condition (NR) (Fig 6A, lanes 5–7), confirming that they were expressed and secreted correctly. As control, Fc portion only was also expressed and purified (Fig 6A, first lane). Again, immunoprecipitates of BEC lysates with scFv-Fc B6-11, but not those treated with Fc only, contained CD146 (Fig 6B), confirming that the fusion antibody retained its capability to bind CD146. As scFv-Fc B6-11 did not detect CD146 in immunoblotting (not shown), it was re-examined in ELISA format, showing that scFv-Fc B6-11 specifically bound to recombinant CD146, but not to control protein (Fig 6C). Further, scFv-Fc B6-11 bound to BECs and melanoma cell lines A375, CRL-1676 and HTB71 harbouring CD146, but not to LECs and HEK-293 cells, in cell ELISA (Fig 6D), and signal intensities correlated with CD146 levels seen in WB (Fig 5D). In immunofluorescence, scFv-Fc B6-11 showed discriminatory labeling of PDPN-negative BECs in HDMECs (Fig 6E, left panel), similar to the commercial anti-CD146 antibody (Fig 6E, right panel). Further, on A375, CRL-1676 and HTB71 melanoma cells, scFv-Fc B6-11 co-stained with an antibody against melanoma surface antigen S-100 (S6A Fig) and with commercial anti-CD146 antibody (S6B Fig). These data confirmed that scFv-Fc antibody B6-11 recognized an antigenic site of CD146, which is common to BECs and melanoma cells.

### scFv-Fc fusion antibodies label BEC membranes

To compare the specificities of scFv-Fc fusion antibodies B6-11, B6-112 and B6-117, co-stainings against endothelial marker CD31were performed on HDMECs. Although there was some variation in the staining intensity, each antibody, but not Fc portion only, re-confirmed the membrane-associated labeling pattern (Fig 6F). A more detailed comparative inspection denoted an intense membrane staining by B6-11 (S7A Fig), while B6-112 showed labeling of the membranous linings (S7B Fig) and B6-117 revealed a more heterogeneous staining pattern (S7C Fig). These subtle differences suggested that the three antibodies recognized diverse cellular epitopes. In conclusion, the data showed that scFv-Fc fusion antibodies, as their scFv parent antibodies, retained reactivity towards antigens presented by BECs in native condition.

### scFv-Fc fusion antibodies label blood vasculature in different organs

**Fig 7:**
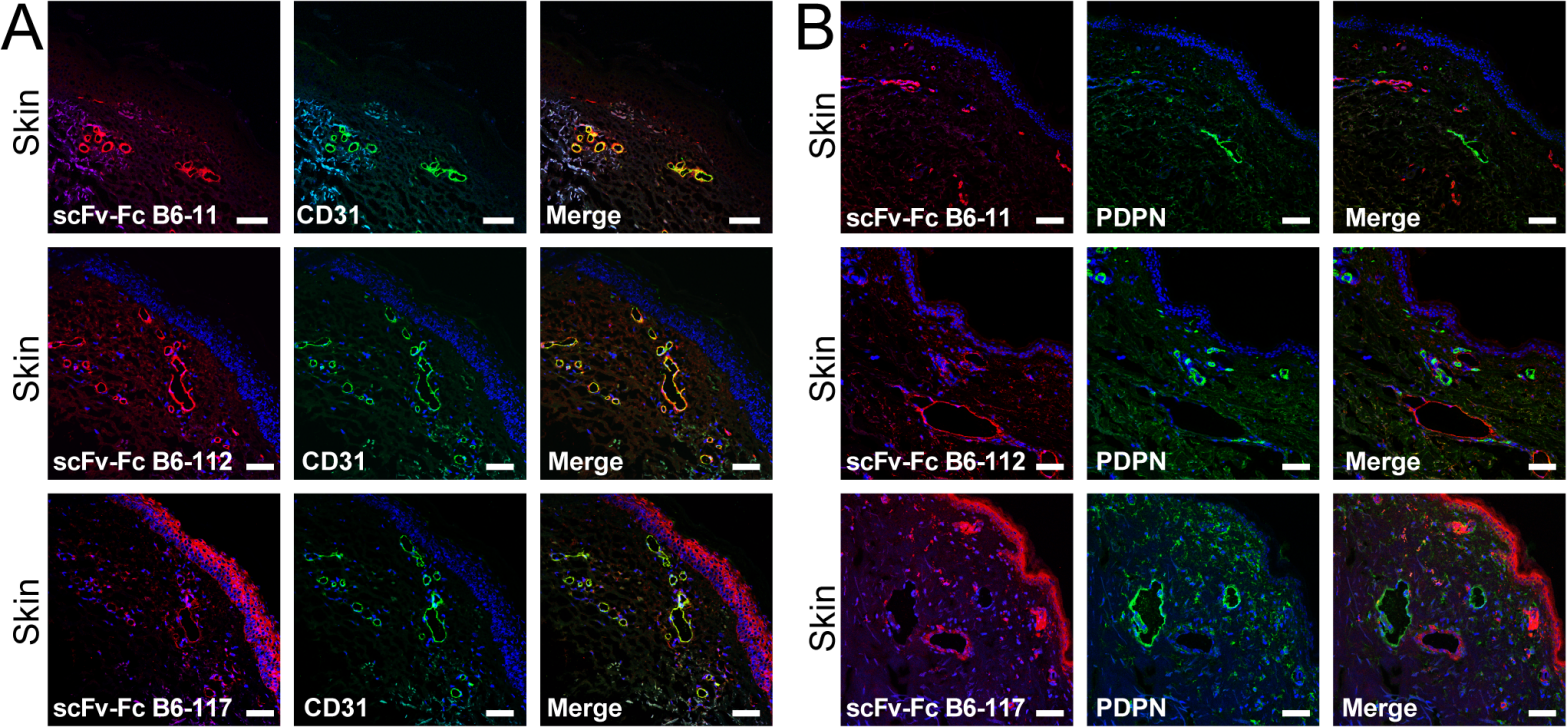
Fusion antibodies scFv-Fc B6-11, B6-112 and B6-117 stain blood vessels in the dermis. A: Representative images of double immunofluorescence stainings of frozen human skin sections with scFv-Fc fusion antibodies (red) in combination with anti-CD31 (green) show overlapping staining, but B: not with anti-PDPN antibody (green) as control. Nuclei were counter-stained with DAPI. Size bars: 50μm.

Next, the binding profile of scFv-Fc fusion antibodies was characterized against a panel of human tissues. As seen by co-localization with endothelial marker CD31 (Fig 7A), but not with lymphatic marker podoplanin (Fig 7B), scFv-Fc B6-11, B6-112 and B6-117 fusion antibodies specifically stained blood vessel structures of human skin sections. Staining of blood vasculature of kidney specimens was seen by scFv-Fc B6-11, B6-112 and B6-117 fusion antibodies (S8A–S8C Fig), but not with Fc portion only (S8D Fig). Moreover, scFv B6-11 showed specific blood vessel labeling on human colon and lung cryosections (S8E and S8F Fig). These data proved that scFv-Fc fusion antibodies were highly useful as specific labels of blood capillaries from different organs.

### scFv-Fc B6-11 interferes with gap closure of immortalized BECs

**Fig 8:**
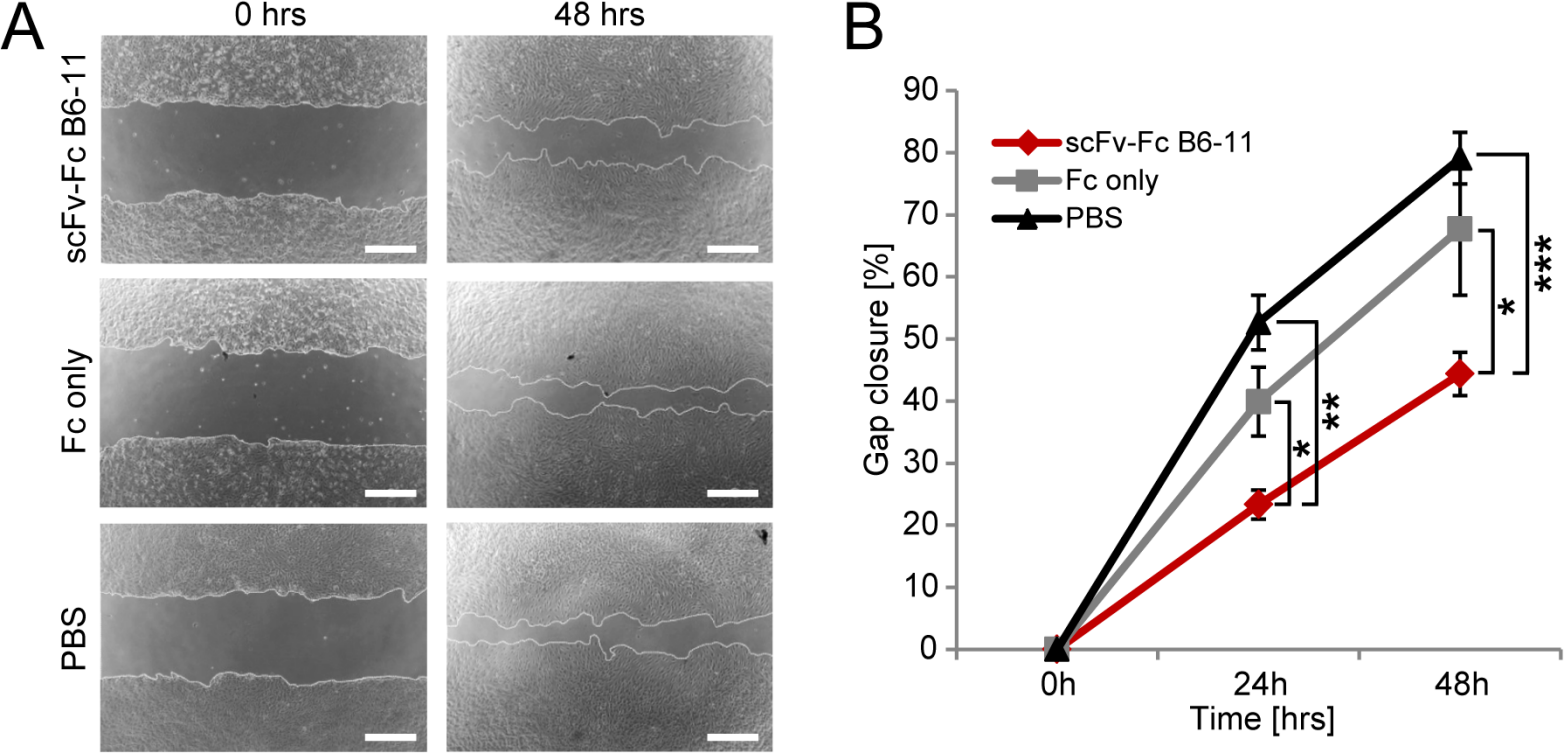
scFv-Fc B6-11 fusion antibody inhibits motility of BECs in a scratch wound assay. A: Migration analysis of BECs by scratch wound assay in the presence of scFv-Fc B6-11, Fc fragment or PBS as controls. Shown are representative images of wounds created in BEC monolayers (n = 3 in each group) at indicated time points. Size bars: 500μm. B: Reoccupation of the gap by BECs after t = 24 hrs and t = 48 hrs versus t = 0 hrs was measured using AxioVision 4.7 software. Areas repopulated by BECs and % of gap area newly covered by BECs were calculated at each timepoint. Assay was performed twice and values are means ± SD. Gap closure was analysed by one-way Anova (*P* < 0.05) followed by pairwise Student´s t-testing. Shown are significant differences between scFv-Fc versus Fc control, and scFv-Fc versus PBS control, respectively (*P*-values: * < 0.05, ** < 0.005, ***< 0.0005).

Finally, considering the putative functional role of CD146 for endothelial cell migration [38], we analyzed whether binding of scFv-Fc B6-11 to CD146 present on BECs showed implications *in vitro*. In a scratch wounding assay, addition of scFv-Fc B6-11 to cell culture medium lead to a significantly decreased gap closure of a BEC monolayer (Fig 8A, upper panel, and Fig 8B, red line), when compared to addition of Fc portion (Fig 8A, middle panel and Fig 8B, grey line) or PBS only as controls (Fig 8A, lower panel, and Fig 8B, black line), suggesting that scFv-Fc B6-11 might exert an inhibitory influence on the migratory capabilities of BECs via binding to CD146.

In conclusion, we show the feasibility of phage antibody enrichment on BECs versus LECs, yielding 166 unique phage antibodies (S9A Fig). Out of these, we isolated three antibodies, B6-11, B6-112 and B6-117, that exclusively target blood vasculature, and by using IP and LC-MS, one antibody specificity was identified as MCAM/CD146 (S9B Fig) revealing an antigenic link between blood endothelial and melanoma cells.

## Discussion

Endothelial cells (ECs) lining blood and lymphatic vessels have become important areas of interest, since it has been recognized that ECs derived from different compartments display diverse molecular features [40,41,42]. Moreover, ECs undergo certain alterations during processes like ageing, obesity, diabetes and malignant disease, e.g. by providing different routes for tumor spread [43]. However, the molecular nature of these subtle differences is only recently being explored and, still, reagents that reliably characterize EC subtypes are needed. Our method was useful to derive a panel of scFv antibodies against membrane-associated BEC and LEC proteins.

By avoiding cell lysis or labelling, we presented native conformations, including post-translational modifications (glycosylation), of endothelial surface proteins to the phage antibodies. Antibody phage display pannings have been performed previously on EC lysates [19,25], on whole ECs [17,20,21,22,44], or on vascular tissue *in vivo* [23,24,26], leading to isolation of reactive probes. The innovative aspect of our approach was the application of phage antibodies on intact cell surfaces of clearly defined human primary BECs and LECs at very low passages and under careful control of specific marker expression.

Similar to other studies [16,17], direct pannings on BEC and LEC monolayers to maintain cell-cell and cell-matrix interactions did not work in our hands, possibly due to reduced antigen availability and / or stickyness of the plastic cell culturing devices [16,45]. Hence, as performed in other studies [34,46,47,48], we conducted enrichments on high cell densities of BECs and LECs freshly harvested and kept in suspension, in order to provide concentrated membrane antigen presentation. As cells express a multitude of potential epitopes at once, selections are largely driven by the density of the target antigens, and we expected that a large number of anti-endothelial antibodies would have to be isolated in order to obtain a representative survey of the EC surface. By extending enrichments over six selection rounds, we aimed to force the selection pressure to its limits. This strategy led to strongly increased EC-specific phage titers, which exceeded those of other studies [17,20], as seen by the high enrichment-factors gained in selection rounds #4 - #6 (Table 1).

FACS binding and *BstN*I fingerprinting pointed at enrichment of functional clones. However, as others observed restricted phage recoveries from cell selections [19,20], we immediately turned to phagemid sequencing prior to antibody binding profiling. Out of nearly 1.000 randomly picked and sequenced clones, we retrieved 557 (56%) intact antibody clones, which lead to 166 (17%) unique sequences. This result is comparable to other studies that have used non-immune or semi-synthetic libraries [26,47,49]. Most of the sequences were unique, while 50 sequences were recovered in multiple frequency from the selections (Table 2), and especially one clone (L5-44) was selection-dominant, emerging in BECs and LEC enriched phages. The fact that we derived binders common to BECs and LECs suggests that the nature of the antigens, rather than the cell-specific background, seem to affect the selection outcome. The emergence of clones with restricted diversity might account for selection against specific ligands, as was reported previously for the use of immunized libraries [21]. The results might indicate that ETH-2 phage antibodies recognize specific selection-dominant epitopes on ECs. The selection should then favour subsets of CDR3 families to be enriched. Homology alignment of the 166 diverse scFv sequences showed no common feature of the VH- and VL-CDR3 sequences (S3 Fig), underlining unbiased enrichment of phage antibodies. Further, VH- and VL-CDR3 regions of single clones descended from diverse branches of the phylogenetic trees, pointing at diversity of the bound antigens.

Gold-standard during detailed phage antibody profiling was specific targeting of BECs and/or LECs, showing that percentages of binding reactivities were comparable to other studies [25,34]. Among the 166 unique scFvs, three classes of anti-EC scFvs were identified: (i) lineage-specific scFvs (BEC versus LEC); (ii) scFvs reactive to BECs and weaker to LECs; and (iii) low reactive scFvs (BEC and LEC). Antibodies that strongly reacted to BECs and weaker to LECs probably bound the same antigen, yet present in lower concentration or abundance on LECs. Among these was also the prominent scFv L5-44 reactive to both, BECs and LECs, which we neglected in further analyses, because we were interested in the retrieval of antigens differential for BECs and LECs.

Successful isolation of several antibodies against recombinant antigens has proved ETH-2 to be a robust and reliable antibody source [29,30,31]. However, it cannot be completely excluded that certain EC antigens preferentially bind to a subset of CDR3 domains covered by the ETH-2 library. The antibodies that reacted equally well with both, BECs and LECs, might recognize antigens common to ECs. Many of these clones did not show eminent cell binding and were neglected for further analyses. However, these low binders might only have weak affinities or bind to EC antigens present in very low concentrations. Still, they probably target interesting ligands, which should be clarified by analyzing further scFvs of our selection.

Originally aiming to derive phage antibodies against both cellular subtypes, we failed to derive LEC-specific entities. Though subtractive screening has been applied to enrich for differentially expressed antigens [50], in our hands, this approach was not successful to derive LEC-specific clones, which might be due to the following reasons: It has been established by transcriptomal and proteomic comparison that, despite expressing several characteristic marker antigens, BECs and LECs represent very close related cell lineages [8]. In addition to recently documented phenotypic EC plasticity [40], some substantial amount of endothelial marker molecules seem to become downregulated during *in vitro* culturing [8,51]. Further, antigen modifications might contribute to altered binding strength: Possibly a substantial difference of the so-called glycocalyx of endothelial cells [52] might be due to the failure of extensive antibody selections on LECs: Distinct levels of surface protein glycosylations could lead to a general steric hindrance which could limit antibody accessibility to the proteinaceous core of the antigens. Use of a phage antibody library pre-immunized with LECs, or direct selections on lymphatic tissue *ex vivo* could probably overcome this limitation. Basically, these uncertainties indicate that further studies on the principal antigenic features of BECs versus LECs, including analysis of their glycoproteomic differences, are necessary.

Detailed antibody validation of eight scFv candidates showed that scFvs detected antigens *in vitro* and on frozen tissue sections, but not on denatured protein material. Direct antibody selection on native antigens has been shown to mainly lead to recognition of native epitopes [13], and conformation-specific antibodies are highly valuable in terms of medical diagnosis and therapeutical research [50]. Three scFvs exclusively stained BECs, and five scFvs labeled both, BECs and LECs, yet exhibiting distinct binding patterns, which suggests diverse antigen recognition. Still, these candidates represent a potentially important class of antibodies for targeting both, blood and lymphatic vessels.

Most importantly, scFv antibodies fully retained their binding effectiveness in phage-free format, and after fusion to a murine Fc portion. Fusion to constant Fc portion might increase binding capacities of scFv antibodies by providing structural support and stabilizing its conformation [53]. The antibodies B6-11, B6-112 and B6-117 bound to targets present on blood versus lymphatic vasculature in skin and kidney, making them interesting candidates for vascular imaging. Further studies might uncover additional antibody clones that recognize specific vascular sites.

Retrieval of CD146/MUC18 as homing target of scFv B6-11 adds to its already known value as a discriminatory blood endothelial marker [54]. Confirmative to this study, we identify CD146 as a highly reliable surface antigen on BECs versus LECs, whose expression is maintained during long term culture. Only few studies have succeeded in *de novo* identification of antigens recognized by antibodies derived from phage-display, and these were obtained from pannings against rodent endothelial cells [24,25,26]. Hence, for the first time, we present a panel of scFvs targeting the molecular repertoire of primary human ECs. At best, we gained scFvs against 166 diverse antigens (S9B Fig). As we aimed to isolate discriminatory antigens, we did not focus on the analysis of high-frequency clones reactive to both cell lines, for example clone L5-44. Due to the fact that immunoprecipitations and subsequent mass-spectrometry are technically demanding and cost-extensive, we could not follow antigen-analysis of further hits. However, we believe that, besides identification of CD146, there are other well-known markers of BECs and/or LECs among these.

Selection of an anti-CD146 scFv could indicate that CD146 preferentially binds to sequences covered by the ETH-2 library. However, scFv B6-11 showed only a frequency of 5 times among the 557 intact sequences, excluding an antigenic bias of the ETH-2 library. On the other hand, we have to consider that screenings on intact cells led to identification of diverse scFvs or Fabs recognizing the same antigen, (1) when libraries immunized against a certain antigen were used [55], or (2) when the antigen of choice was overexpressed [48]. Probably some of our clones recognize different epitopes of the same antigens, which can be shown by further antigen / epitope mapping.

There are concerns that glycosylation limits accessibility of phage antibodies to the proteinaceous core of potential antigens [16]. However, the anti-CD146 scFv B6-11 was fully capable to react with the peptide core of CD146 even after removal of N-linked glycosylation, indicating that BECs and LECs were not over-protected by a glycocalyx that made their epitopes inaccessible. Overall, successful isolation of phage antibodies on intact cells seems to depend either on the occurrence of selection-dominant epitopes, or on the abundance of surface antigens. Also here, further clones might be evaluated to identify the antigenic diversity of our antibody panel.

The fact that scFv B6-11 recognizes CD146 expressed by BECs and melanoma cells indicates the potential of phage display technology to identify molecular markers common for different cell types. It is known that tumors upregulate functional membrane adhesion molecules for enhanced growth and invasiveness [56]. CD146 was identified as an endothelial junction protein involved in the control of cell-cell cohesion and cell migration [38]. Co-expression of CD146 by BECs and tumor cells supports this notion, indicating functional analogies between diverse cellular entities. Hence, by using antibody phage display, it may be possible to identify antigenic associations with pathologies, and B6-11 might be useful to study upregulation of CD146 on the surface of diverse malignant cell lines and tissues [57].

Preliminary data indicated that B6-11 antibody might interfere with migratory capacity of BECs (Fig 8). There is baseline level expression of CD146 in every vascular bed [54], and, until now, there is no evidence that CD146 is overexpressed in tumor blood vessels. However, though dispensable during embryonic vessel development [58], CD146 has been shown to be required for tumor neo-angiogenesis as a co-receptor of VEGFR-2 during VEGF-induced pro-angiogenic signaling [38,59]. Hence, the authors of these studies suggest anti-CD146 therapy as a probable adjunct option to anti-VEGF treatment. Previous studies have isolated antibodies against CD146 [60], among these function blocking candidates [61], and a peptide-phage panning approach yielded two epitope-mimotopes that interfered with antibody binding [62]. Similar to other anti-CD146 antibodies [63,64,65], future studies might show whether B6-11 exerts anti-angiogenic capabilities, probably identifying a homing motif which enables specific anti-angiogenic targeting.

The vasculature is an attractive target for imaging and therapy, as the antigens are accessible and should be bound rapidly, allowing fast antibody residence. Antibody B6-11 might be useful for vascular imaging to trace enhanced microvessel density in tumors, and, subsequently, could be important for diagnostic applications. Previously, phage-derived peptides have been used as homing and blocking motifs to tumor blood vessels [66,67]. A number of recent publications highlight the usefulness of antibody phage display to select for potentially therapeutic candidates [28,68], and phage derived human scFvs have been shown to successfully interfere with angiogenesis [14,69,70,71]. Further detailed analyses (e.g. by Biacore) might evaluate whether the antibodies are of high enough affinity to be applicable as reagents for immunodiagnostics. When necessary, multivalent antibody fragments may be used and offer improved functional properties over free scFvs.

## Conclusions

Here, we succeeded in the production of a panel of phage antibodies against human primary BEC and LEC surfaces. The screening translated into successful hits validated through imaging, showing that BECs and LECs share a majority of surface antigens, which is complemented by cell-type specific, unique markers. Our antibody panel can be used as a research tool to further characterize EC antigens, and to investigate possible links with pathological conditions.

## Supporting Information

S1 Fig Early passages of HDMECs contain equal amounts of BEC and LEC subpopulations. Identity control of cultured human dermal HDMECs used for isolation of BECs and LECs for *in vitro* cell panning experiments. Double-immunofluorescence stainings of combinations of endothelial marker molecules show that HDMECs contain equal subpopulations of CD31+/CD44+/VE-Cadherin+ BECs (red) and PDPN+/LYVE-1+ LECs (green). Size bars: 50μm.
(TIF)

S2 Fig Identical amounts of phage preparations used for selections on BECs and LECs. ELISA of ETH-2 library and VCS-M13 wildtype phage dilution series. In addition to titer determinations, dilutions of phage preparations were coated and detected in phage amount ELISA. Sigmoid signal curves confirm comparable amounts of ETH-2 library and VCS-M13 wildtype phages, which were subsequently used for biopannings on BECs and LECs.
(TIF)

S3 Fig Phylogenetic analysis of CDR3 regions of 166 diverse scFv antibodies derived from sequencing (S3 Table), based on alignment of the amino acid sequences. A: Phylogenetic tree of VH CDR3 regions (aa 95–100). B: Phylogenetic tree of VL CDR3 regions (aa 91–96). Alignment trees were built by using CLC Main Workbench 7 software, applying the following settings: alignments were established with a gap open cost of 100 and a gap extension cost of 10, and trees were then constructed with the neighbour joining method and Junkes contor as measure for protein distance. Boxes and inscriptions indicate the twelve scFv clones that were subsequently analysed more detailed.
(TIF)

S4 Fig Results of Mascot alignment with peptides derived from MS/MS analysis of immunoprecipitates with clone scFv B6-11. Shown are retrieved peptides found in Swissprot and in NCBI databases. The same peptides are depicted in Fig 5A, showing alignment to the amino acid sequence of CD146.
(TIF)

S5 Fig scFv B6-11 fusion to Fc in pFUSE6-Fc expression vector. A: Schematic representation of IgG, scFv and scFv-Fc structures. B: Scheme of pFUSE-mIgG2B-Fc2 immunoglobulin expression vector. scFv inserts were PCR-amplified and cloned into the pFUSE expression vector, leading to fusion with murine Fcγ portion. The pFUSE expression vector contains an IL2 secretion signal, which provides secretion of scFv-Fc antibodies by transfected mammalian cells. C: Amino acid sequence of scFv B6-11. Region beween asterisks was cloned into pFUSE mIgG2B. Red letters: Variable amino acid sequence within VH CDR3 und VL CDR3 regions (see also S2 Table). Boxed letters: linker region.
(TIF)

S6 Fig scFv-Fc B6-11 fusion antibody co-localizes with CD146 expressed by melanoma cell lines A375, CRL1676 and HTB71. A: Representative images of double immunofluorescence stainings of A375, CRL1676 and HTB71 melanoma cells with scFv-Fc B6-11 (red) and anti-S100 antibody (green) as positive control. B: Representative images of double immunofluorescence stainings of A375, CRL1676 and HTB71 melanoma cells with scFv-Fc B6-11 (red) in combination with anti-CD146 antibody (green) as positive control. Nuclei were counterstained with DAPI (blue). Size bars: 50μm.
(TIF)

S7 Fig scFv-Fc antibodies B6-11, B6-112 and B6-117 reveal diverse staining specificities. Representative images of double immunofluorescence stainings of HDMECs with A: scFv-Fc B6-11, B: B6-112 and C: B6-117 (red) in combination with CD31 (green). Nuclei were counterstained with DAPI (blue). Size bars: 20μm.
(TIF)

S8 Fig scFv-Fc antibodies B6-11, B6-112 and B6-117 specifically stain blood vessels in kidney, colon and lung. A-C: Representative images of double immunofluorescence stainings of human frozen kidney sections showing a glomerulus and an adjacent blood vessel with scFv-Fc fusion antibodies (red) and anti-CD31 antibody (green) as control. D: Negative control: incubation with Fc fragment only. E: Co-localization of scFv-Fc B6-11 (red) with CD31 (green) in capillaries of human colon cryosections. F: Co-localization of scFv-Fc B6-11 (red) with CD31 (green) in capillaries of human lung cryosections. Nuclei were counterstained with DAPI (blue). Size bars: 50μm.
(TIF)

S9 Fig Summary of phage antibody selection process and subsequent scFv antibody profiling analyses. A: Scheme of selection process of scFv phage antibodies on human dermal BECs and LECs. After enrichment on BECs and LECs, 994 phage clones randomly picked from panning rounds #5 and #6 were sequenced to identify 557 intact scFv sequences (56%). Out of these, 166 unique scFvs were derived. B: Schematic representation of subsequent scFv antibody specificity profiling procedure. 166 unique scFv antibodies were screened in cell ELISA, yielding 86 (53%) antibodies strongly binding to BECs and LECs. Out of these, 12 highly reactive phage scFv antibodies were further analyzed. 8 scFvs showing strongest affinity were expressed without phages, revealing that 3 of these were specific for BECs. These were fused to Fc portion and characterized more detailed. Finally, the antigenic target of one antibody was identified.
(TIF)

S1 Materials and Methods Human Dermal Fibroblast (HDF) isolation and cultivation. Primary Normal Human Dermal Fibroblasts (HDF) were generated during the separation of HDMECS into LECs and BECs as CD31-/PDPN- cell fraction. For culturing, Dulbecco's Modified Eagle Medium (DMEM) with 1% Pen/Strep solution supplemented with 10% fetal calf serum (FCS) was used. HDFs were grown in an incubator at 37°C and 5% CO_2_.
(DOCX)

S1 Table Primary and secondary antibodies used for *ELISA: enzyme linked immunoadsorbent assay; †MB: magnetic bead sorting; ‡IF: Immunofluorescence; #FACS: Fluorescence-activated cell sorting; ||WB: Western blotting. (DOCX)

S2 Table Summary of sequence characteristics of 166 different scFv antibodies directed against human BECs and LECs. *VH and VL CDR3 DNA stretches and †deduced amino acid sequences using respective numbering [32] are given. ‡Sequences were sorted according to occurrence in the initial pool of 557 intact clones. Underlined letters indicate antibody clones analyzed more detailed in this study.
(DOCX)

S3 Table VH and VL CDR3 insert length abundance of scFv clones. ^a^ Amino acid lengths of retrieved VH and VL CDR3 sequences; ^b^ Occurence of CDR3s with respective amino acid lengths in 166 retrieved diverse scFv antibody clones (for detailed sequences see S2 Table).
(DOCX)
