# Supplementary figures and images for: Selection of scFv Antibody Fragments Binding to Human Blood versus Lymphatic Endothelial Surface Antigens by Direct Cell Phage Display
Source: PLoS One. 2015 May 20;10(5):e0127169. doi: 10.1371/journal.pone.0127169 (PMC4439027; doi:10.1371/journal.pone.0127169)

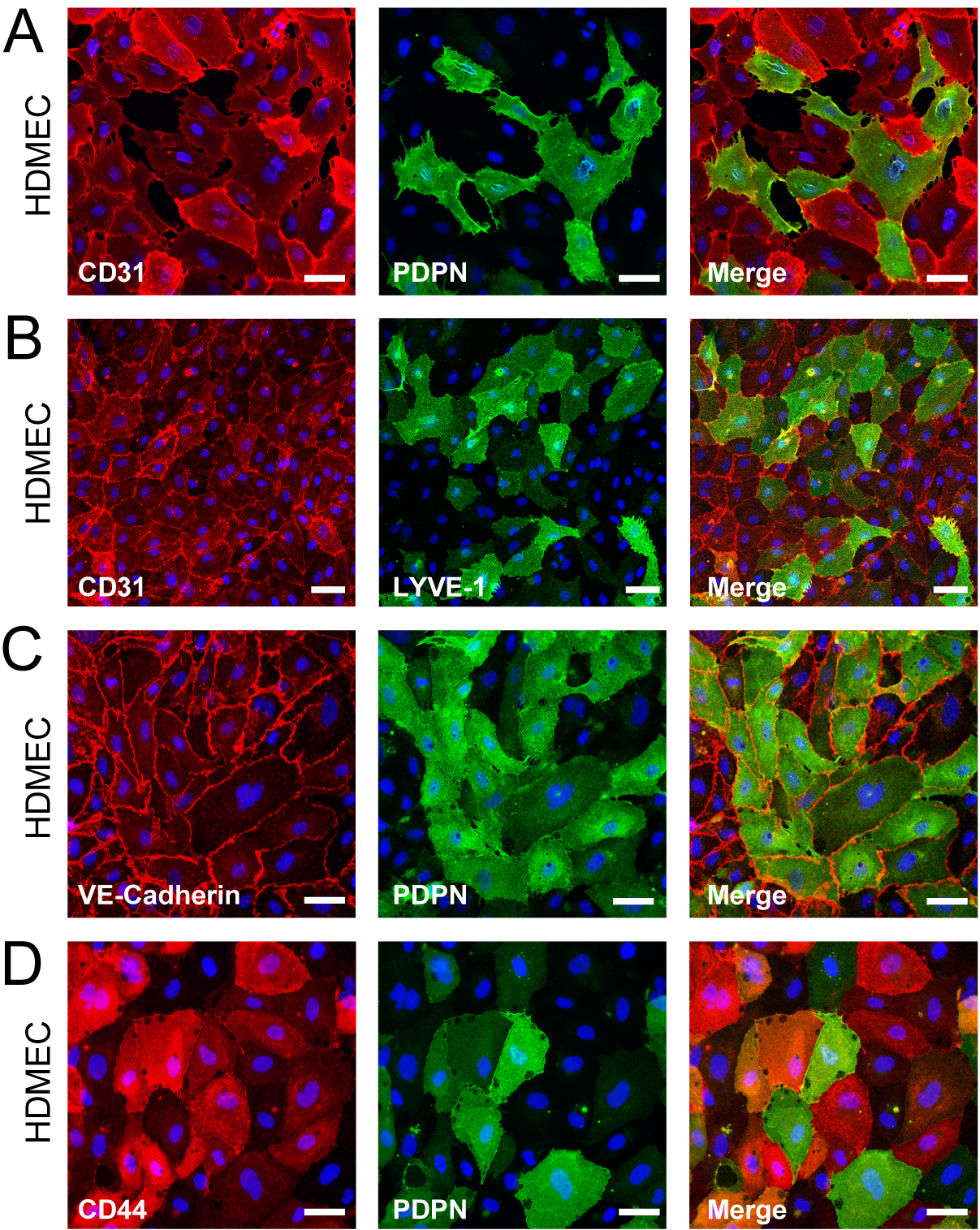

Supplement: S1 Fig — Identity control of cultured human dermal HDMECs used for isolation of BECs and LECs for in vitro cell panning experiments. Double-immunofluorescence stainings of combinations of endothelial marker molecules show that HDMECs contain equal subpopulations of CD31+/CD44+/VE-Cadherin+ BECs (red) and PDPN+/LYVE-1+ LECs (green). Size bars: 50μm. (TIF) [file pone.0127169.s001.tif]

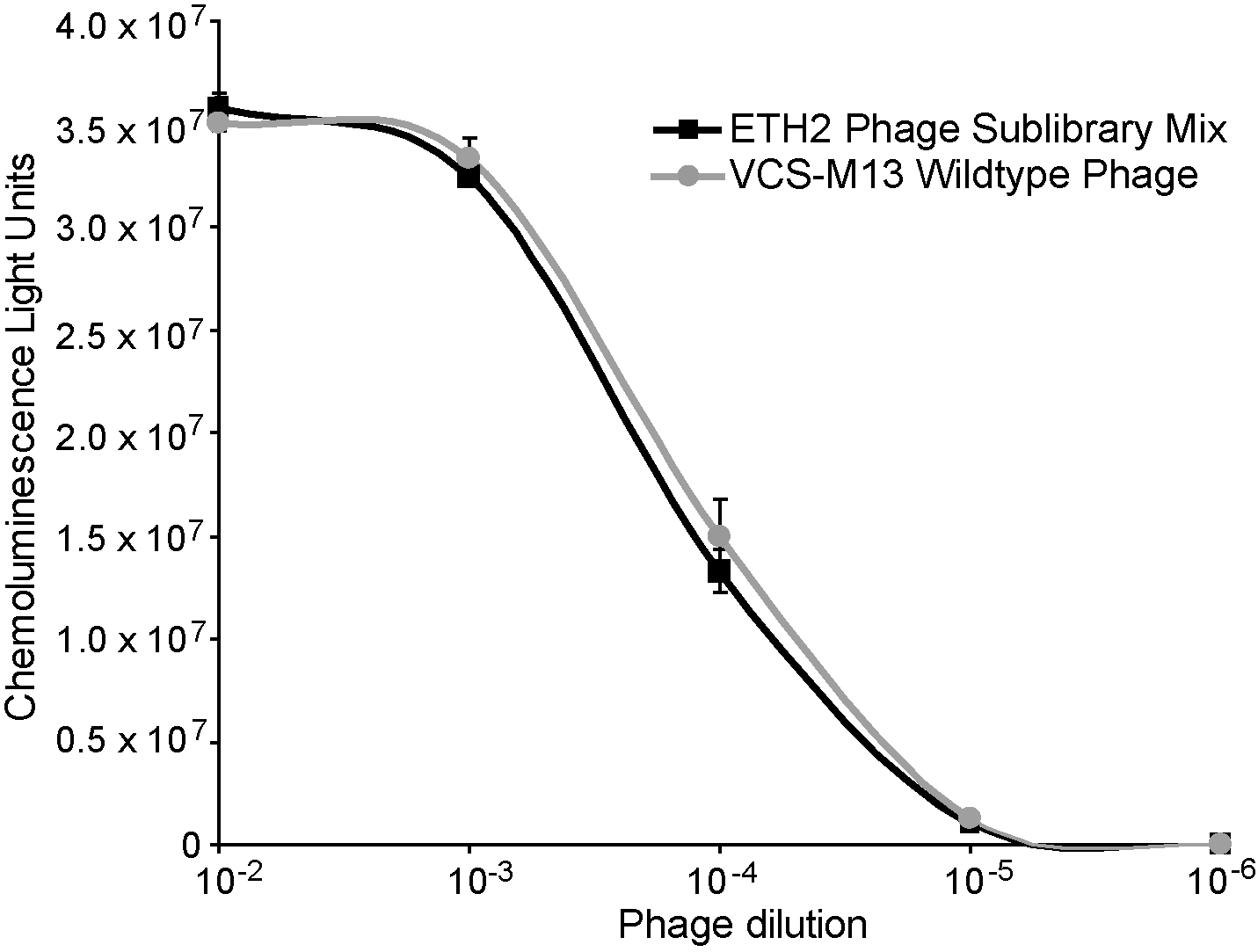

Supplement: S2 Fig — In addition to titer determinations, dilutions of phage preparations were coated and detected in phage amount ELISA. Sigmoid signal curves confirm comparable amounts of ETH-2 library and VCS-M13 wildtype phages, which were subsequently used for biopannings on BECs and LECs. (TIF) [file pone.0127169.s002.tif]

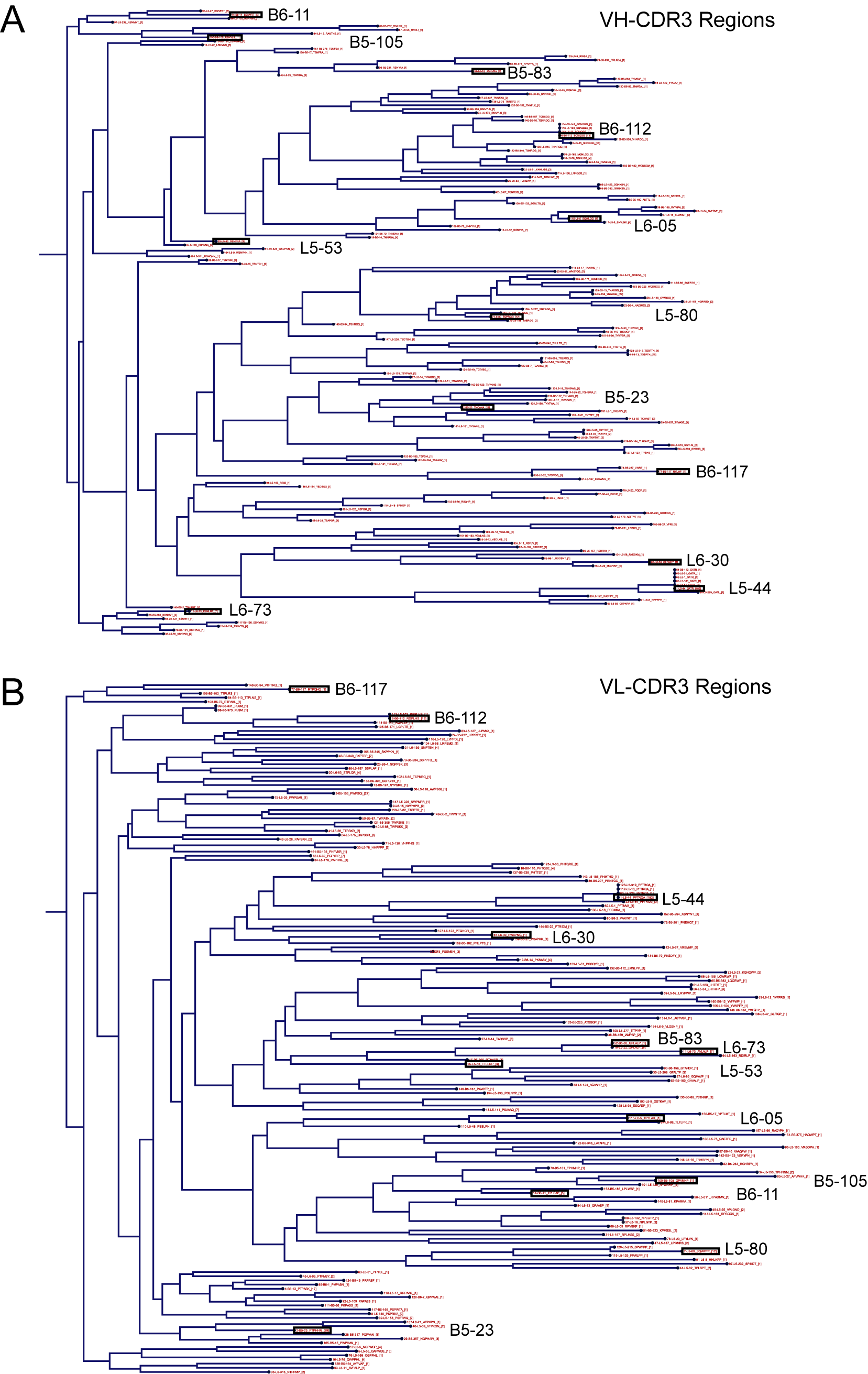

Supplement: S3 Fig — A: Phylogenetic tree of VH CDR3 regions (aa 95–100). B: Phylogenetic tree of VL CDR3 regions (aa 91–96). Alignment trees were built by using CLC Main Workbench 7 software, applying the following settings: alignments were established with a gap open cost of 100 and a gap extension cost of 10, and trees were then constructed with the neighbour joining method and Junkes contor as measure for protein distance. Boxes and inscriptions indicate the twelve scFv clones that were subsequently analysed more detailed. (TIF) [file pone.0127169.s003.tif]

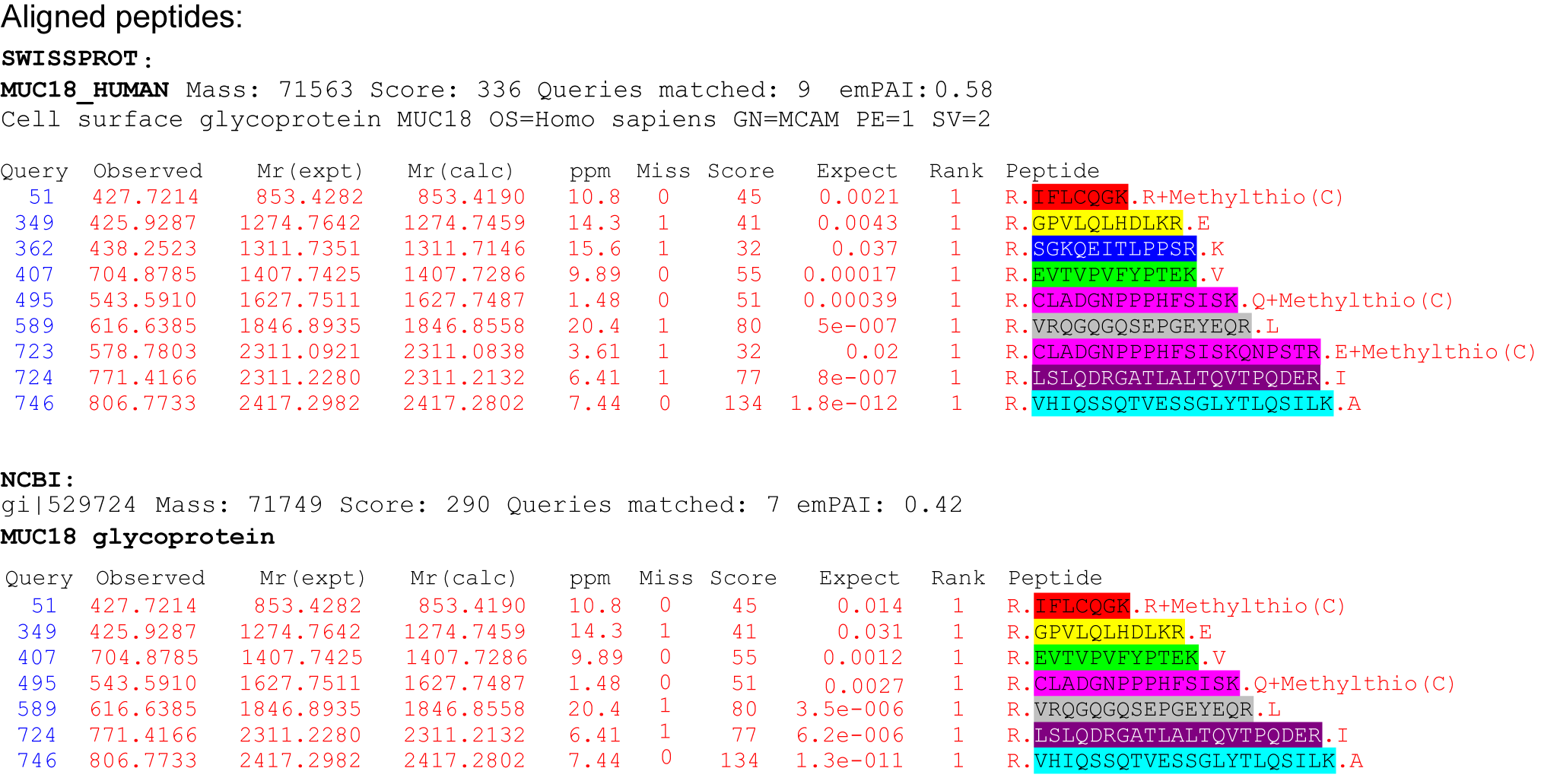

Supplement: S4 Fig — Shown are retrieved peptides found in Swissprot and in NCBI databases. The same peptides are depicted in Fig 5A, showing alignment to the amino acid sequence of CD146. (TIF) [file pone.0127169.s004.tif]

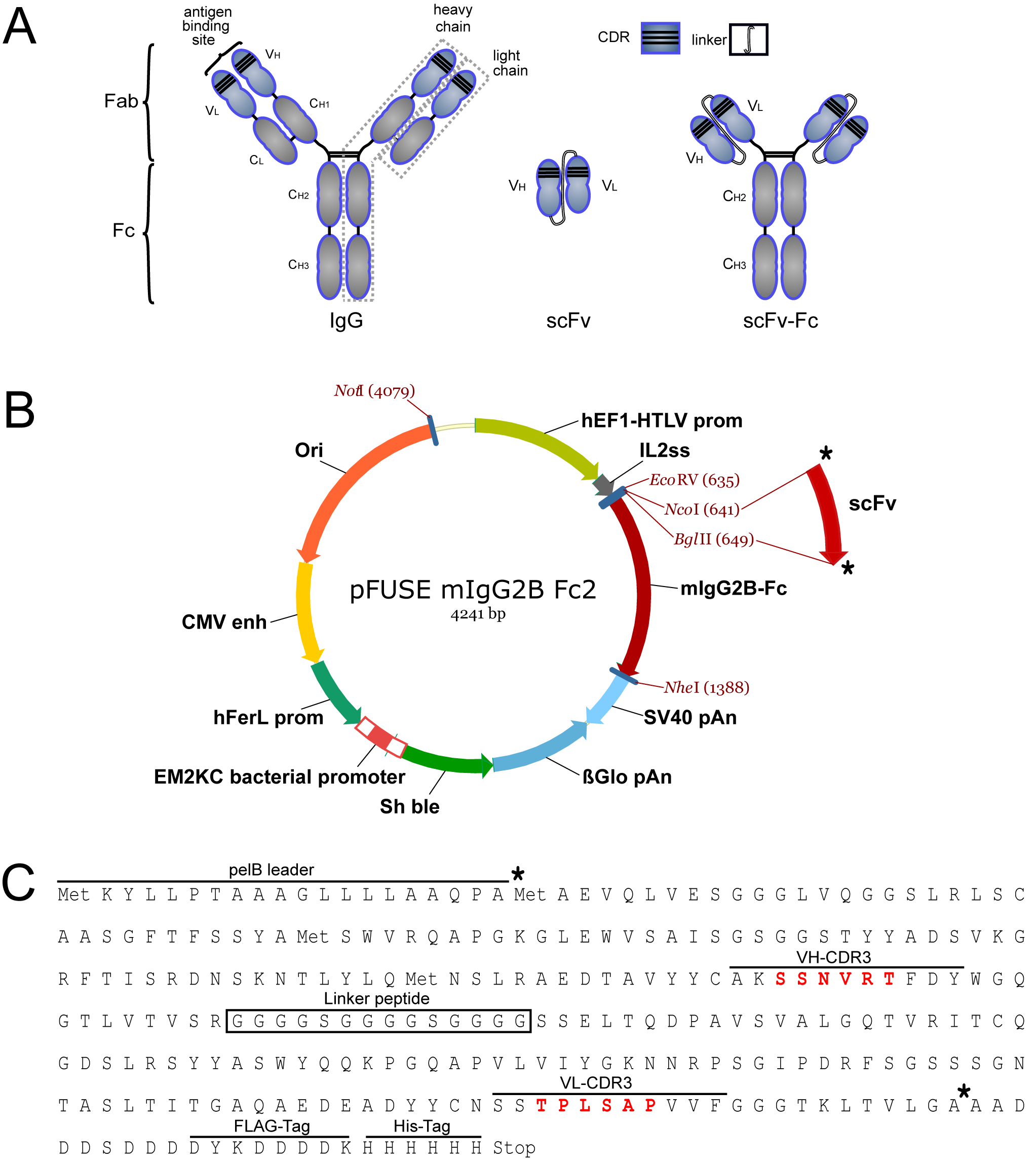

Supplement: S5 Fig — A: Schematic representation of IgG, scFv and scFv-Fc structures. B: Scheme of pFUSE-mIgG2B-Fc2 immunoglobulin expression vector. scFv inserts were PCR-amplified and cloned into the pFUSE expression vector, leading to fusion with murine Fcγ portion. The pFUSE expression vector contains an IL2 secretion signal, which provides secretion of scFv-Fc antibodies by transfected mammalian cells. C: Amino acid sequence of scFv B6-11. Region beween asterisks was cloned into pFUSE mIgG2B. Red letters: Variable amino acid sequence within VH CDR3 und VL CDR3 regions (see also S2 Table). Boxed letters: linker region. (TIF) [file pone.0127169.s005.tif]

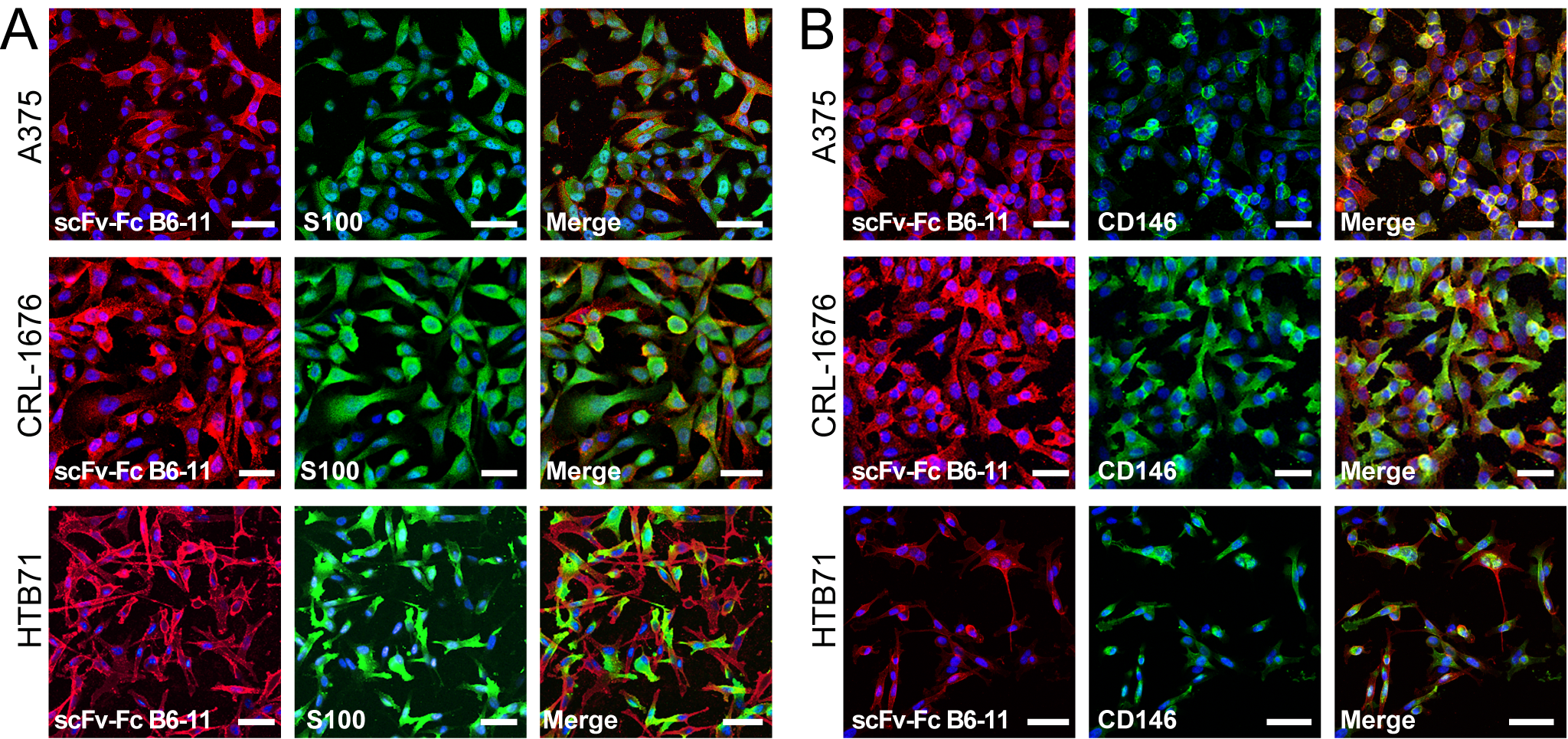

Supplement: S6 Fig — A: Representative images of double immunofluorescence stainings of A375, CRL1676 and HTB71 melanoma cells with scFv-Fc B6-11 (red) and anti-S100 antibody (green) as positive control. B: Representative images of double immunofluorescence stainings of A375, CRL1676 and HTB71 melanoma cells with scFv-Fc B6-11 (red) in combination with anti-CD146 antibody (green) as positive control. Nuclei were counterstained with DAPI (blue). Size bars: 50μm. (TIF) [file pone.0127169.s006.tif]

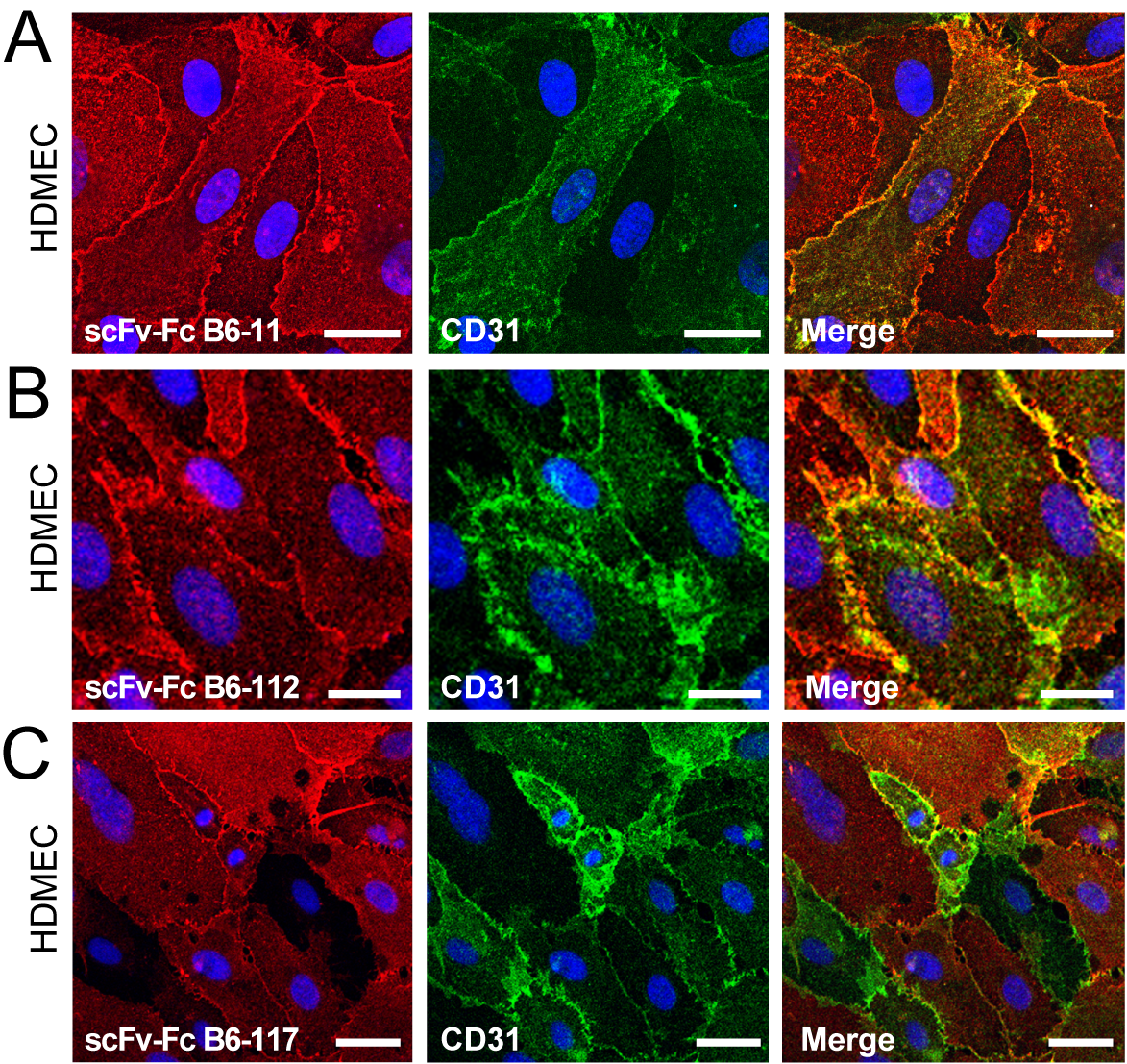

Supplement: S7 Fig — Representative images of double immunofluorescence stainings of HDMECs with A: scFv-Fc B6-11, B: B6-112 and C: B6-117 (red) in combination with CD31 (green). Nuclei were counterstained with DAPI (blue). Size bars: 20μm. (TIF) [file pone.0127169.s007.tif]

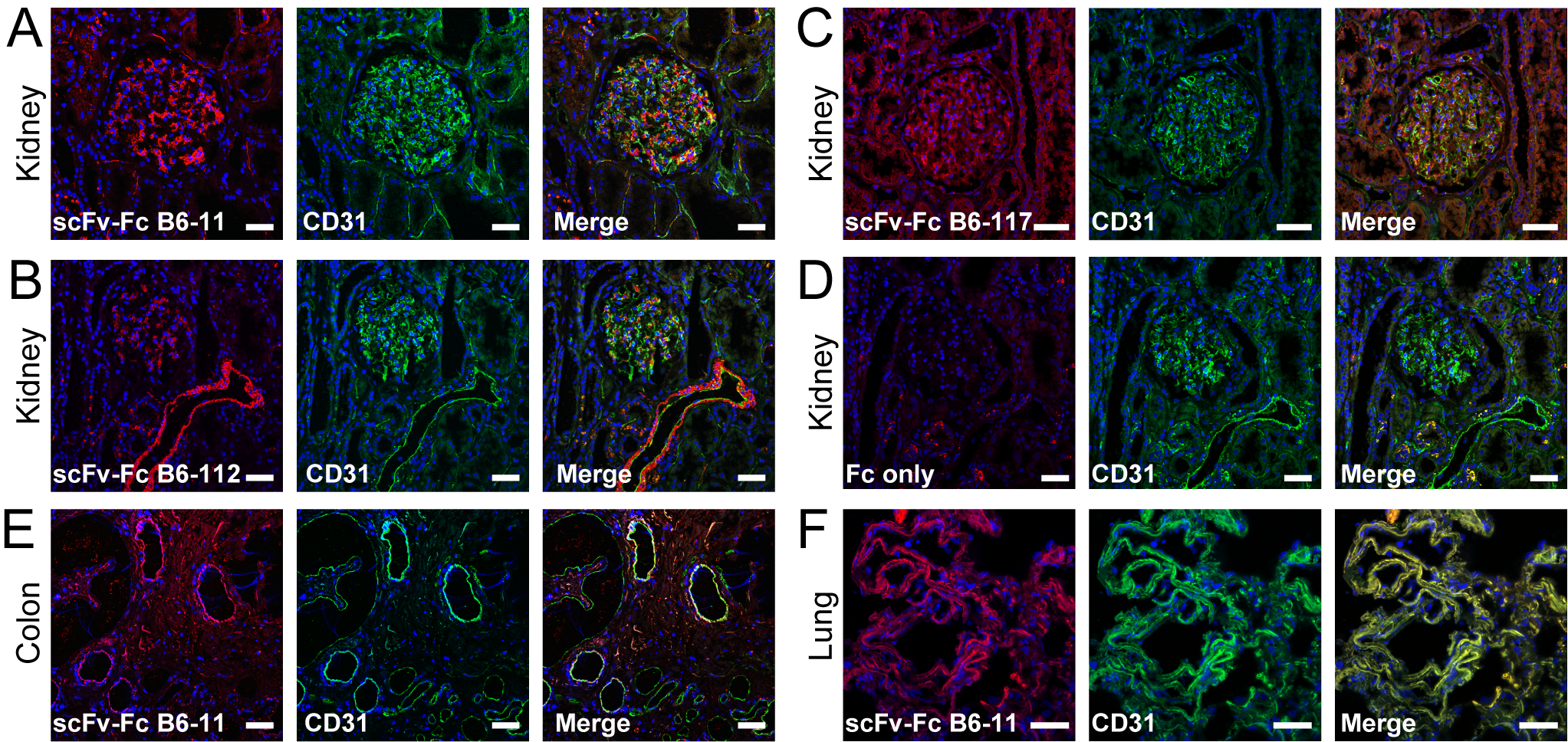

Supplement: S8 Fig — A-C: Representative images of double immunofluorescence stainings of human frozen kidney sections showing a glomerulus and an adjacent blood vessel with scFv-Fc fusion antibodies (red) and anti-CD31 antibody (green) as control. D: Negative control: incubation with Fc fragment only. E: Co-localization of scFv-Fc B6-11 (red) with CD31 (green) in capillaries of human colon cryosections. F: Co-localization of scFv-Fc B6-11 (red) with CD31 (green) in capillaries of human lung cryosections. Nuclei were counterstained with DAPI (blue). Size bars: 50μm. (TIF) [file pone.0127169.s008.tif]

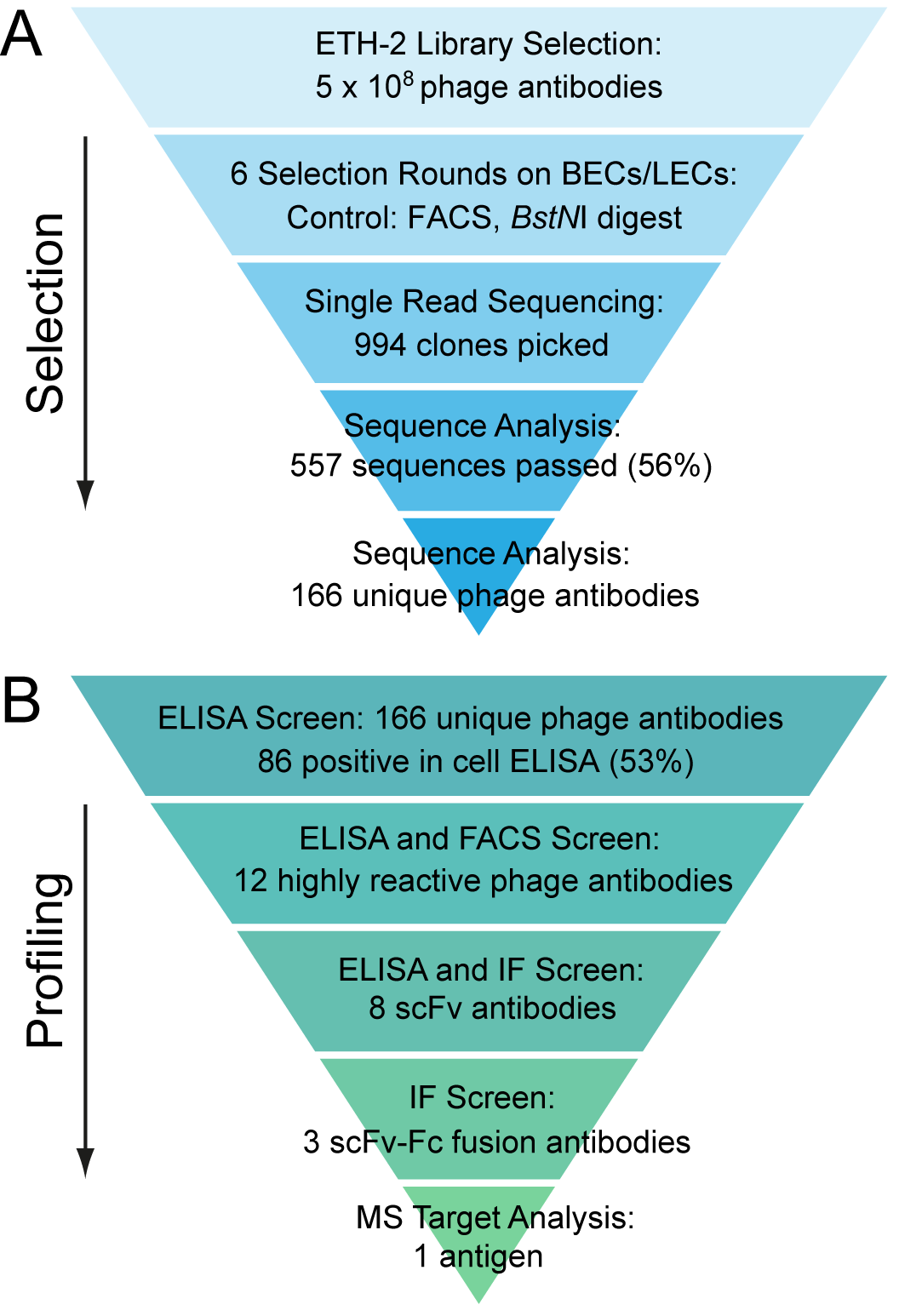

Supplement: S9 Fig — A: Scheme of selection process of scFv phage antibodies on human dermal BECs and LECs. After enrichment on BECs and LECs, 994 phage clones randomly picked from panning rounds #5 and #6 were sequenced to identify 557 intact scFv sequences (56%). Out of these, 166 unique scFvs were derived. B: Schematic representation of subsequent scFv antibody specificity profiling procedure. 166 unique scFv antibodies were screened in cell ELISA, yielding 86 (53%) antibodies strongly binding to BECs and LECs. Out of these, 12 highly reactive phage scFv antibodies were further analyzed. 8 scFvs showing strongest affinity were expressed without phages, revealing that 3 of these were specific for BECs. These were fused to Fc portion and characterized more detailed. Finally, the antigenic target of one antibody was identified. (TIF) [file pone.0127169.s009.tif]
