# Supplementary material for: Selection of scFv Antibody Fragments Binding to Human Blood versus Lymphatic Endothelial Surface Antigens by Direct Cell Phage Display
Source: PLoS One. 2015 May 20;10(5):e0127169. doi: 10.1371/journal.pone.0127169 (PMC4439027; doi:10.1371/journal.pone.0127169)
Supplement: S1 Materials and Methods — Primary Normal Human Dermal Fibroblasts (HDF) were generated during the separation of HDMECS into LECs and BECs as CD31-/PDPN- cell fraction. For culturing, Dulbecco's Modified Eagle Medium (DMEM) with 1% Pen/Strep solution supplemented with 10% fetal calf serum (FCS) was used. HDFs were grown in an incubator at 37°C and 5% CO2. (DOCX) [file pone.0127169.s010.docx]

**S1 Material and Methods**

**Human Dermal Fibroblast (HDF) isolation and cultivation**

Primary Normal Human Dermal Fibroblasts (NHDF) were generated during the separation of HDMECS into LECs and BECs as CD31-/PDPN- cell fraction. For culturing, Dulbecco's Modified Eagle Medium (DMEM) with 1% Pen/Strep solution supplemented with 10% fetal calf serum (FCS) was used. HDFs were grown in an incubator at 37°C and 5% CO_2_.
