# Supplementary material for: Selection of scFv Antibody Fragments Binding to Human Blood versus Lymphatic Endothelial Surface Antigens by Direct Cell Phage Display
Source: PLoS One. 2015 May 20;10(5):e0127169. doi: 10.1371/journal.pone.0127169 (PMC4439027; doi:10.1371/journal.pone.0127169)
Supplement: S1 Table — (DOCX) [file pone.0127169.s011.docx]

**S1 Table**

| **Antibody Description** | **Application** | **Cat. Number** | **Company / Source** | **Dilution** |
| --- | --- | --- | --- | --- |
| anti-M13, HRP-conjugate, mouse monoclonal | *ELISA | 27-9421-01 | GE Healthcare | 1:1000 - 1:3000  for ELISA |
| anti-penta-His, HRP-conjugate, mouse monoclonal | ELISA | 34460 | Qiagen | 1:2000 |
| anti-fd bacteriophage rabbit polyclonal | ELISA | B7786 | Sigma | 1:800 |
| anti-CD31(clone JC70A), mouse monoclonal | ^†^MB, ^‡^IF | M0823 | DAKO Cytomation | 1:50 for MB+IF  1:200 for ELISA |
| anti-podoplanin antiserum, rabbit, IgG fraction (6.4mg/ml) | ^#^FACS, MB,  IF, ^\|\|^WB, ELISA |  | in house developed | 1:150 for FACS 1:500 for MB+IF 1:3000 for WB  1:1000 for ELISA |
| anti-CD44, mouse monoclonal | IF | MS-178-PABX | Neo Markers | 1:100 |
| anti-Lyve-1, rabbit polyclonal | IF | 11-032 | Angiobio | 1:100 |
| anti-VE-Cadherin, mouse monoclonal | IF | PNIM-1597 | Beckman Coulter | 1:100 |
| anti-CD146, rabbit monoclonal | IF, WB, ELISA | EPR3208 | Abcam | 1:1000 for IF 1:1000 for WB  1:100 for ELISA |
| anti-CD146/MUC18 (AA98), mouse monoclonal (2A5) | ELISA | IM1010 | Calbiochem | 1:500 |
| anti-S100, rabbit polyclonal | IF | ab76729 | Abcam | 1:100 |
| Alexa Fluor 488 anti-His tag, clone 4D11, mouse monoclonal | IF, FACS | 16-254 | Millipore | 1:500 for IF  1:200 for FACS |
| Alexa Fluor 488 anti-Penta-His tag, mouse monoclonal | IF | 35310 | Qiagen | 1:1000 |
| Alexa Fluor 546 F(ab')2 fragment of goat anti-mouse IgG (H+L) | IF | A-11018 | Molecular Probes | 1:1000 |
| Alexa Fluor 488 goat anti-rabbit IgG (H+L), highly cross-adsorbed | IF | A-11034 | Molecular Probes | 1:1000 |
| Alexa Fluor 546 goat anti-rabbit IgG (H+L) | IF | A-11010 | Molecular Probes | 1:1000 |
| Alexa Fluor 488 F(ab')2 fragment of goat anti-mouse IgG (H+L) | IF | A-11017 | Molecular Probes | 1:1000 |
| Alexa Fluor 488 donkey anti-mouse IgG (H+L) | IF | A-21202 | Molecular Probes | 1:1000 |
| Alexa Fluor 594 donkey anti-goat IgG (H+L) | IF | A-11058 | Molecular Probes | 1:1000 |
| anti-FLAG tag M2, mouse monoclonal | IF | F3165 | Sigma-Aldrich | 1:1000 |
| HRP-conjugated rabbit anti-mouse antibody | ELISA, WB | JZM035046 | Axell | 1:3000 for ELISA  1:3500 for WB |
| HRP-conjugated goat anti.rabbit antibody | ELISA, WB | SGZ034047 | Axell | 1:3000 for ELISA  1:3500 for WB |
