# Supplementary material for: Selection of scFv Antibody Fragments Binding to Human Blood versus Lymphatic Endothelial Surface Antigens by Direct Cell Phage Display
Source: PLoS One. 2015 May 20;10(5):e0127169. doi: 10.1371/journal.pone.0127169 (PMC4439027; doi:10.1371/journal.pone.0127169)
Supplement: S2 Table — *VH and VL CDR3 DNA stretches and †deduced amino acid sequences using respective numbering [32] are given. ‡Sequences were sorted according to occurrence in the initial pool of 557 intact clones. Underlined letters indicate antibody clones analyzed more detailed in this study. (DOCX) [file pone.0127169.s012.docx]

**S2 Table**

| Nr. | Clone | *VH CDR3 sequence between TGTGCGAAA……..TTTGACTAC | ^†^VH CDR3  95 – 100 | *VL CDR3 sequence between AACTCCTCT……..GTGGTATTC | ^†^VL CDR3  91 – 96 | ^‡^Frequency |  |
| --- | --- | --- | --- | --- | --- | --- | --- |
|  |  |  |  |  |  |  |  |
| 1 | L5-44 | GGGGCGACTCGG | G A T R | CCCTTTACTAGGCAGGCG | P F T R Q A | 182 | |
| 2 | B5-23 | ACTAATCAGGCTAATGCG | T N Q A N A | CCCACTCCGCATCATAAT | P T P H H N | 29 | |
| 3 | B5-156 | TATAATGCGCGTGGGGGG | Y N A R G G | CCCTGGCCGTCGCAGATT | P W P S Q I | 27 | |
| 4 | B6-13 | ACGGGTAGTTTTACGAAT | T G S F T N | CCGACGCCCGCTTCTAAG | P T P A S K | 17 | |
| 5 | L5-55 | TCGCATAATCGTGGGGGG | S H N R G G | CAGGCGCCCTGGGGGTCT | Q A P W G S | 13 | |
| 6 | B6-112 | TCGCAGAATCAGGGGGGG | S Q N Q G G | CGGGGTCCCCTTCGTTCG | R G P L H S | 13 | |
| 7 | L5-80 | ACTCAGCCGAGGGGGGGT | T Q P R G G | TCTCAGTGGTTTCCGCCC | S Q W F P P | 12 | |
| 8 | L5-149 | TCGAGTAATTATAATGCT | S S N Y N A | CCCTCGCCTCGGAAGGCG | P S P R K A | 9 | |
| 9 | L6-15 | ACGAGTAATACGGGGCAT | T S N T G H | AATTGGCCGATGCCCCGG | N W P **M** P R | 9 | |
| 10 | L5-22 | TTGTCGAATATGAATAGT | L S N **M** N S | CAGCCGTTGGCGCTGCCC | Q P L A L P | 8 | |
| 11 | L6-73 | AAGTCTAATTTGAATACG | K S N L N T | GCGGTTTTGGCTCTTCCC | A V L A L P | 7 | |
| 12 | L5-32 | TCTGGGAATACTGTTGCT | S G N T V A | CCTGGTCCGTATCGTCCC | P G P Y R P | 7 | |
| 13 | L5-141 | ACGAGTCATGCGAATGCA | T S H A N A | CCCAGTGCTGCGGCGCAG | P S A A A Q | 7 | |
| 14 | B6-11 | AGTTCGAATGTGCGTACT | S S N V R T | ACTCCTCTTTCGGCGCCC | T P L S A P | 5 | |
| 15 | B5-366 | AAGTCGAATTATAATACG | K S N Y N T | CGTACTATGGCGAGTCCC | R T **M** A S P | 4 | |
| 16 | L5-76 | ATGGGGAATCTGGGGGGT | M G N L G G | CAGTGGCCCCCTCATCTG | Q W P P H L | 4 | |
| 17 | L5-05 | AGTAATGTGCTTAATTTT | S N V L N F | AATGGTCCCTGGGGTCCG | N G P W G P | 4 | |
| 18 | B6-110 | ACTCATGATGTGGGTCCT | T H D V G P | CCCCATACGCAGAGTGAG | P H T Q S E | 4 | |
| 19 | B6-14 | ACTAATAATGCTAATGCG | T N N A N A | CCCAAGAGTGCTTCTTAT | P K S A S Y | 4 | |
| 20 | L6-83 | ACTCAGAATAGTTGGGCC | T Q N S W A | TCGACGCCCTTGCAGCGT | S T P L Q R | 4 | |
| 21 | L5-139 | ACTTCTAATTATACTGGG | T S N Y T G | TCTAATCCCACGGAGAAT | S N P T E N | 4 | |
| 22 | L5-84 | GGGGCGACTCGG | G A T R | CCCTTTACTAGGCAGGGG | P F T R Q G | 3 | |
| 23 | B6-04 | AATGCTGATCGGGGTGGG | N A D R G G | TCGCAGCCCCCGTCTAAG | S Q P P S K | 3 | |
| 24 | L5-175 | TCGAATAATTATCTTGGT | S N N Y L G | CAGGCTCCCTCTTCGAGG | Q A P S S R | 3 | |
| 25 | L5-53 | TCTAGTAATTTTAATGCG | S S N F N A | ACTACGCTGTTGCGTCCC | T T L L R P | 3 | |
| 26 | L5-34 | TCGGTTTTTGGTGTTGAG | S V F G V E | TTGCATACTCGGTTTCCC | L H T R F P | 3 | |
| 27 | L6-14 | ACGAATTGGCAGGGAAGT | T N W Q G S | ACGGCGCAGTCGTCTCCC | T A Q S S P | 3 | |
| 28 | B5-317 | ACGAGTAATACGAATAAC | T S N T N N | CCTCAGCCCGTGGCGAAT | P Q P V A N | 3 | |
| 29 | B5-357 | ACGTATATGGCGTCTGAG | T Y **M** A S E | AATCAGCCCCATGCGTGG | N Q P H A W | 3 | |
| 30 | L6-75 | TGGGGGAATTATGCTCTT | W G N Y A L | CCCTCGAGTATGTCTAAT | P S S M S N | 3 | |
| 31 | L5-167 | ATTGGGTGGTGGAATGGG | I G W W N G | AGGCCCTTGCATTCTAGT | R P L H S S | 2 | |
| 32 | L5-21 | AAGAATAATCTGGGGGGG | K N N L G G | AAGGGTAATCAGTGGCCC | K G N Q W P | 2 | |
| 33 | L5-78 | AAGTCTAATTATAATAGT | K S N Y N S | CATCATCCGTTTCCGCCC | H H P F P P | 2 | |
| 34 | L5-153 | AATGGTAGGAGGGAGGAT | N G R R E D | ACTCCCCATAATAATATG | T P H N N **M** | 2 | |
| 35 | L5-268 | TCGATTTATTCTCATAGT | S I Y S H S | GGGTTTGCGCTTACGCCC | G F A L T P | 2 | |
| 36 | L5-316 | TCGATTTATACGCATTCG | S I Y T H S | AATACTCCCCCTATGCCG | N T P P **M** P | 2 | |
| 37 | L6-16 | TCTTTGTGGATGCAGACT | S L W **M** Q T | AATCCTCTGGGGACTCCC | N P L G T P | 2 | |
| 38 | B6-159 | AGTGTGTATATGGCTGCT | S V Y **M** A A | ATTGCTATGCCGGCTCCC | I A M P A P | 2 | |
| 39 | L5-158 | ACGGCGGAGAGGGGGGGT | T A E R G G | CCCAGTCCTACGTGGCAG | P S P T W Q | 2 | |
| 40 | L5-88 | ACGGGGCTGAAGTCTGGT | T G L K S G | ACTTGGCCCTCGAAGAAG | T W P S K K | 2 | |
| 41 | L5-28 | ACTGGTAATCTTTGGCCT | T G N L W P | ACGACGCCCTCGAAGCGG | T T P S K R | 2 | |
| 42 | L5-67 | ACTGGTAATAGGTCGGGT | T G N R S G | GTTCGTTCGATGATGCCC | V R S **M** **M** P | 2 | |
| 43 | B5-340 | ACGCATCTGTTGACGGGT | T H L L T G | TCGAAGCCCACGTCTCCC | S K P T S P | 2 | |
| 44 | L5-82 | ACGAAGTGGGCTTCGACT | T K W A S T | ACTCCCCTTAGTCCGACG | T P L S P T | 2 | |
| 45 | L6-99 | ACTAAGTGGACGCATACT | T K W T H T | CCCACGCCTATGAGTTAT | P T P **M** S Y | 2 | |
| 46 | L5-39 | ACTAAGTATACTCATACT | T K Y T H T | GTGACGCCCAAGGGTAAT | V T P K G N | 2 | |
| 47 | L5-137 | ACGAATAATTTTGCTGGG | T N N F A G | CTGCCCGGGATGCGTAGT | L P G **M** R S | 2 | |
| 48 | L6-28 | ACGTCTGCTCCTGGTCCT | T S A P G P | CCCGCTCCTTCGAAGAAT | P A P S K N | 2 | |
| 49 | L5-25 | ACTTCTAATTATCGGGCT | T S N Y R A | GTGCCCTTGGGGAATGAT | V P L G N D | 2 | |
| 50 | B5-87 | TGGAATAGTACTGGTGGG | W N S T G G | ACGTGGCCCGCGACGAAT | T W P A T N | 2 | |
| 51 | B5-323 | TGGAGTGATTTTAATAAT | W S D F N N | AAGCCCATGTCTTCGCTT | K P M S S L | 2 | |
| 52 | B5-83 | GCTGATAATTATCGTGCG | A D N Y R A | CAGCCCCTTGCGCTTCCG | Q P L A L P | 1 | |
| 53 | L6-12 | GCGAGTGATTTGCATTCT | A S D L H S | TATGTTTTTCCGAGGTCC | Y V F P R S | 1 | |
| 54 | L5-178 | GCGTCGTTTCCTTATACG | A S F P Y T | CCCGCTCCGCATCGGCTT | P A P H R L | 1 | |
| 55 | B5-160 | GCGTCGACTACTTTG | A S T T L | GGTCATGCTGCGCTTCCC | G H A A L P | 1 | |
| 56 | L5-118 | TGTTATGCTCGTGGGGGG | C Y A R G G | GCCTGGCCGTCTGGGATT | A W P S G I | 1 | |
| 57 | B6-40 | GATTGGTATCCT | D W Y P | GTTGCGGCGCAGCCCTGG | V A A Q P W | 1 | |
| 58 | L5-124 | GAGAGTAATTATAATACA | E S N Y N T | GCTGGGGCGGCTCGTCCC | A G A A R P | 1 | |
| 59 | L5-52 | TTTGGGAATTTGGGTAGC | F G N L G S | CTGAGGTATCCGTGGCCC | L R Y P W P | 1 | |
| 60 | L5-229 | GGGGCGACTCTG | G A T L | CCCTTTACTAGGCAGGCG | P F T R Q A | 1 | |
| 61 | L5-183 | GGGGCGACTCGG | G A T R | TTGCATACTCGGTTTCCC | L H T R F P | 1 | |
| 62 | L5-01 | GGGGCGACTCGG | G A T R | CCCTTTACTATGGTGGCG | P F T **M** V A | 1 | |
| 63 | L6-51 | GGGGCGACTCGG | G A T R | CCCATTCCCACGTCTGAG | P I P T S E | 1 | |
| 64 | B6-113 | GGGGCGACTCGG | G A T R | ACGACGCCCCTTAATAGT | T T P L N S | 1 | |
| 65 | B5-383 | GGTGGTAATAAGTCGAAT | G G N K S N | CTGCAGTGTAGGTGGCCC | L Q C R W P | 1 | |
| 66 | L5-155 | GGGGGTAATAAGTCGAAT | G G N K S N | CTGCAGTGGAGGTGGCCC | L Q W R W P | 1 | |
| 67 | L6-88 | GGTAAGCCTAATCCTGCT | G K P N P A | ACGCTTACTTTGCCCCGT | T L T L P R | 1 | |
| 68 | L5-132 | ATCTTTGTGGATGCAGACT | I F V D A D | AACCCTTTGGGAACTCCC | N P L G T P | 1 | |
| 69 | L6-05 | AAGAATAATACTGCTGAG | K N N T A E | AGGCCGGTTGGGAAGCCC | R P V G K P | 1 | |
| 70 | B5-101 | AAGTCGAATTATAATGGG | K S N Y N G | ACTCCCCATATGCATCCG | T P H **M** H P | 1 | |
| 71 | L5-138 | CTGAATAATCAGGGTAGT | L N N Q G S | GTGCATCCCTTTCATGGT | V H P F H G | 1 | |
| 72 | B5-201 | TTGCCTGATCATTCT | L P D H S | CCCAATGAGCATCAAACG | P N E H Q T | 1 | |
| 73 | B5-124 | CTGAGTAATCAGAATGCA | L S N Q N A | TCGTATCCCAGTCGGGAG | S Y P S R E | 1 | |
| 74 | B5-237 | CTTTGGCGGACT | L W R T | TTGCCCCCTCGTGAGTAT | L P P R E Y | 1 | |
| 75 | L5-29 | ATGGGTGATGTGAAGCCT | M G D V K P | CCCTGGCCGAGTGCTAGG | P W P S A R | 1 | |
| 76 | L5-169 | ATGGGGAATCTGGGGGGT | M G N L G G | CAGGGGCCCCCTCATCTG | Q G P P H L | 1 | |
| 77 | B6-117 | AATTATCTGTGG | N Y L W F | GGACGCCCCAGAATCAG | R T P Q N Q | 1 | |
| 78 | L5-20 | CCGCAGGAGCCG | P Q E P | TTGCCCTATTTGGTTAAT | L P Y L V N | 1 | |
| 79 | B5-234 | CCTCGGCTTAAGGAGGCT | P R L K E A | CCCCCGACTCAGGTGCAT | S S P P T Q | 1 | |
| 80 | B6-02 | CCTAGTTGTATTCCG | P S C I P | CCCGCGCGGTATCGGACT | P A R Y R T | 1 | |
| 81 | L6-30 | CAGCTTGATAATAAGTAT | Q L D N K Y | CCCTGGGCTCCTAATGGT | P W A P N G | 1 | |
| 82 | B5-263 | CAGCGGATGCCGGGGGTG | Q R **M** P G V | CATGGGCATAGGCCCGTG | H G H R P V | 1 | |
| 83 | L5-127 | AGGGCTTGTCCTCCGACG | R A C P P T | CTGCTTCCCATGTATGCG | L L P **M** Y A | 1 | |
| 84 | L6-13 | CGGGCGAATACGAATTCG | R A N T N S | CAGCCCGCGGCTGAGCCG | Q P A A E P | 1 | |
| 85 | B6-01 | CGTGATGATTCTAATACT | R D D S N T | CCCATGCCGGCGAGTAAT | P **M** P A S N | 1 | |
| 86 | L5-157 | CGTGAGCATCGGGCGTAT | R E H R A Y | TCGTCGCCGCTTGCTCCC | S S P L A P | 1 | |
| 87 | L6-95 | CGTTTTAATCTTATT | R F N L I | GGGCAGATGGCGGTTCCC | G Q M A V P | 1 | |
| 88 | B5-373 | CGTTTTAATTATTTTGCT | R F N Y F A | CCCCTTTCGATG | P L S M | 1 | |
| 89 | B5-207 | CGTAATCTGAGGAGGT | R N L R R | CCCCGGAATACGGGGTGT | P R N T G C | 1 | |
| 90 | B6-156 | AGGAATAATTATTTGAGT | R N N Y L S | GGGACGGCGTTTGATCCC | G T A F D P | 1 | |
| 91 | L6-08 | AGGCCTCCGGAGCCGCAT | R P P E P H | CATCATTTGAAGCCCCCT | H H L K P P | 1 | |
| 92 | L5-109 | CGGAGTGATTTTGCGGTT | R S D F A V | CCCGCGCCTGCTGAGAGT | P A P A E S | 1 | |
| 93 | L5-11 | AGGTCTTTTTTGGTACG | R S F L V | GCTGTTCCGGCTCTTCCC | A V P A L P | 1 | |
| 94 | L5-163 | CGGTCTATTGGT | R S I G | AGGGATATTCGTTTGCCC | R D I R L P | 1 | |
| 95 | L5-27 | CGTAGTAATTTTAGGACT | R S N F R T | GCTCCCGTGTGGCATAAG | A P V W H K | 1 | |
| 96 | L5-100 | CGGTCTAATCATATGACA | R S N H **M** T | GTTCGGGGGGATCCCGCT | V R G D P A | 1 | |
| 97 | L5-239 | CGTTCTAATATGAATACT | R S N **M** N T | AGTCCCATTAAGGATACT | S P I K D T | 1 | |
| 98 | L5-511 | CGTAGTAATCAGAATAAT | R S N Q N N | CGGCCCGCTGATATGTGG | R P A D M W | 1 | |
| 99 | B5-331 | CGTTCGAATTATTTTGCT | R S N Y F A | CCCCTTTCGATG | P L S **M** | 1 | |
| 100 | B5-105 | AGGTCTAATTATTTGAGT | R S N Y L S | GGGCCTGTGAATCATCCC | G P V N H P | 1 | |
| 101 | L5-126 | CGGAGTCCGGGTATG | R S P G **M** | GCTCCCTGGAATCATCCG | A P W N H P | 1 | |
| 102 | L6-86 | CGGTCGCAGCATCCG | R S Q H P | ACGTCTCCCATGCGTGGT | T S P M R G | 1 | |
| 103 | L5-09 | CGTTGGTCGGCT | R W S A | GGGTCGACGAAGGCTCCC | G S T K A P | 1 | |
| 104 | L5-58 | AGGTATAAGTCGAAGATG | R Y K S K **M** | CTGAGGCCCAGTATGGAT | L R P S M D | 1 | |
| 105 | B5-171 | AGTGGGATGCGGGGTGGT | S G **M** R G G | TTGGGTCCCCTGACTGAG | L G P L T E | 1 | |
| 106 | B5-102 | TCTGGGAATTTGACTAGT | S G N L T S | ACGACGCCCCTTCGTTCG | T T P L R S | 1 | |
| 107 | L6-21 | TCGAAGATTCGTGGGGGG | S K I R G G | GCTACGCCGAAGCCGAAT | A T P K P N | 1 | |
| 108 | B5-73 | TCTAATAATTATACGGCG | S N N Y T A | CGGACGCCCGCTAAGCTT | R T P A K L | 1 | |
| 109 | L5-277 | AGTAATCCTAGGGGGGGC | S N P R G G | ACTATTACGCCTTATCCC | T I T P Y P | 1 | |
| 110 | L5-48 | TCGCCTATGGGTCCT | S P **M** G P | CCCAGTAGTCTGCCTCAT | P S S L P H | 1 | |
| 111 | B5-86 | AGTCAGGAGCGTACGGGG | S Q E R T G | CCCAAGCCTGCGTCTTCG | P K P A S S | 1 | |
| 112 | L5-13 | TCGCAGAATCAGGGGGGG | S Q N Q G G | CCCTTTACTAGGCAGGCG | P F T R Q A | 1 | |
| 113 | L5-103 | TCGCAGAATCAGGGGGGG | S Q N Q G G | CGGGGTCCCCTTCATTCG | R G P L H S | 1 | |
| 114 | B5-141 | TCGCAGAATCAGGGGGGG | S Q N Q G G | CGGGGTCCCCTTTCATTCG | R G P L S F | 1 | |
| 115 | L6-05 | TCGCAGTGGCTGGGGGGC | S Q W L G G | ACTCCCACGTTGGCTCAT | T P T L A H | 1 | |
| 116 | L5-120 | AGTCGGCCGGAGACTCTT | S R P E T L | CTGTATCCCCCGGATATT | L Y P P D I | 1 | |
| 117 | B5-166 | TCTAGTAATTATCATGGT | S S N Y H G | CCTTCGCCTTGGACCGCG | P S P W T A | 1 | |
| 118 | L5-17 | ACGGCGGCTACGATGGGT | T A A T **M** G | AGGCGGCCCGCTGCGAGT | R R P A A S | 1 | |
| 119 | L5-129 | ACTGAGCCTAGGGGGGGT | T E P R G G | TTTCCTTGGTTGCCTCCC | F P W L P P | 1 | |
| 120 | B6-07 | ACGGGTGCTCGTAGTGGT | T G A R S G | CAGCCTCCCGCGGTGTCT | Q P P A V S | 1 | |
| 121 | B5-305 | ACGGGGCTGAAGTCTGGT | T G L K S G | ACCTGGCCCTCGAAGGAG | T W P S K E | 1 | |
| 122 | B5-346 | ACTGGGAATAGGGGTGGG | T G N R G G | TTGGCGACGGCTCCCTCG | L A T A P S | 1 | |
| 123 | L5-319 | ACGGGTAGTTTTACGAAT | T G S F T N | CCCTTTACTAGGCAGGCG | P F T R Q A | 1 | |
| 124 | B5-49 | ACGGGTACTTATTCGGGT | T G T Y S G | CCGCGTCCCGCTTCGTTT | P R P A S F | 1 | |
| 125 | L5-30 | ACTCATGATGTGGGTTGT | T H D V G C | CCCCATACGCAGAGGGAG | P H T Q R E | 1 | |
| 126 | L5-215 | ACTCATAATCGGGGGGGG | T H N R G G | TCTCCGTGGTTTCCGCCT | S P W F P P | 1 | |
| 127 | L5-123 | ACTATTTATTCGCATAGT | T I Y S H S | CCCACTCAGCATGGGCGT | P T Q H G R | 1 | |
| 128 | L5-95 | ACGAAGTATACGCATACT | T K Y T H T | GAGTCTCAGGCTGAGCCC | E S Q A E P | 1 | |
| 129 | B5-164 | ACGCTTCATGGTCATAC | T L H G H T | GCGTATCCGGTGGCGCCC | A Y P V A P | 1 | |
| 130 | B6-88 | ACGATGTGGTCTGCGCTT | T M W S A L | TATTCTACGAATAATCCC | Y S T N N P | 1 | |
| 131 | L6-01 | ACGAATGATGCTTATGTT | T N D A Y V | GCGGGGACTGTTGGGCCC | A G T V G P | 1 | |
| 132 | B5-112 | ACTAATCATGCTTGGGCT | T N H A W A | CTTATGAATTTGCCGCCC | L **M** N L P P | 1 | |
| 133 | L5-18 | ACGAATCATTCGTGGTCT | T N H S W S | CCCGAGGGTATGAAGGCG | P E G **M** K A | 1 | |
| 134 | B6-70 | ACTAATAATGATAATGCG | T N N D N A | CCCAAGAGTGATTTTTAT | P K S D F Y | 1 | |
| 135 | B6-152 | ACTAATAATTTTCTGAAG | T N N F L K | TATATGTTTCAGACGCCC | Y **M** F Q T P | 1 | |
| 136 | L5-75 | ACGAATAATACTTTTGGT | T N N T F G | CAGGCTTCCACTCCCAGG | Q A S T P R | 1 | |
| 137 | B5-238 | ACGAATGTGGGGGCTCCT | T N V G A P | CCCCATACTACGAGTACG | P H T T S T | 1 | |
| 138 | L5-47 | ACGAATTGGGCTTGGAGT | T N W A W S | GGTTTGTTTATTGGGCCC | G L F I G P | 1 | |
| 139 | L5-51 | ACTAATTGGCAGAATTCG | T N W Q N S | CCCCAGTCTCAGTATCGT | P Q S Q Y R | 1 | |
| 140 | L6-81 | ACGAATTATGCGGAGACT | T N Y A E T | AAGCCCGCGCGTAAGGCG | K P A R K A | 1 | |
| 141 | L5-161 | ACTAATTATGCTCGGGGT | T N Y A R G | CGTCCCTCTGGTCAGAAG | R P S G Q K | 1 | |
| 142 | B5-123 | ACGAATTATAATAATAGT | T N Y N N S | GTTGGGCGTTATCCCAAT | V G R Y P N | 1 | |
| 143 | L5-186 | ACTAATTATACGTGGGCG | T N Y T W A | CCCCATATGACGCATGAT | P H **M** T H D | 1 | |
| 144 | B5-22 | ACGCAGCATTCGTGGGCT | T Q H S W A | CCCACGCGTATTGATATG | P T R I D **M** | 1 | |
| 145 | B5-16 | ACTCAGAATCGGGGGGGT | T Q N R G G | ACTCGTCATCGGCCCAAT | T R H R P N | 1 | |
| 146 | B5-157 | ACGCAGAATTCGGGGGGG | T Q N S G G | CCTGGGGCGTATACGCCC | P G A Y T P | 1 | |
| 147 | L5-226 | ACGAGTGATACTGGGCAT | T S D T G H | AATTGGCCGATGCCCCGG | N W P **M** P R | 1 | |
| 148 | B5-94 | ACGTCTCATCGGGGGGGC | T S H R G G | GTTACGCCCACGCGTCAG | V T P T R Q | 1 | |
| 149 | B5-02 | ACTTCGAATGCTAATACG | T S N A N T | ACTCCTCCCAATACGCCT | T P P N T P | 1 | |
| 150 | B5-17 | ACGTCTAATTTTAGGGCT | T S N F R A | TATCCCACGTTGATGACG | Y P T L **M** T | 1 | |
| 151 | B5-375 | ACTTCGAATTTTTCGGCG | T S N F S A | CATGCTCAGTGGCCCACT | H A Q W P T | 1 | |
| 152 | B5-294 | ACGTCTCCTGCTAATGTT | T S P A N V | AAGTCGAATTATAATACG | K S N Y N T | 1 | |
| 153 | B5-186 | ACGTCTCCTTCTAATATT | T S P S N I | CTGCCTCTTTGGGCTCCC | L P L W A P | 1 | |
| 154 | L5-133 | ACTTCTTATTTTTGGGGT | T S Y F W G | CCCGGGCTGAATTATCCT | P G L N Y P | 1 | |
| 155 | B5-345 | ACTACTATTGATACTGGG | T T I D T G | TCTAAGCCCCCGAAGAAT | S K P P K N | 1 | |
| 156 | L6-62 | ACTTATGGTTGGGGGGGG | T Y G W G G | ACGGCTCCCCCTACTAGG | T A P P T R | 1 | |
| 157 | L6-96 | ACTTATAAGACGGGTCGG | T Y K T G R | AGGGCGCAGTATCCCCAT | R A Q Y P H | 1 | |
| 158 | B5-308 | GTGCATAATAGGGGTGGT | V H N R G G | AGTTCTCCCGGGAGGCGG | S S P G R R | 1 | |
| 159 | B6-27 | GTTCCTCGTATT | V P R I | CCCCAGGCTCCGAAGAAG | P Q A P K K | 1 | |
| 160 | B6-12 | GTGAGTGATTTGCATTCT | V S D L H S | TATGTTTTTCCGTGGCCC | Y V F P W P | 1 | |
| 161 | B5-193 | GTTAGTATGCTGAATAGT | V S **M** L N S | CCGCATCCCGTGAAGCGT | P H P V K R | 1 | |
| 162 | B5-182 | TGGGGTAATTCTGGTATG | W G N S G **M** | CCCAATCTTCCTACTTCG | P N L P T S | 1 | |
| 163 | B5-225 | TGGCAGGATCGGGGGGGG | W Q D R G G | GCTACGGGGTCTGGGCCC | A T G S G P | 1 | |
| 164 | L6-09 | TGGAGTAATTATAATAAT | W S N Y N N | GTTCTGGGGTCGAATCCC | V L G S N P | 1 | |
| 165 | B5-15 | TATAATGCGCGTGGGGGG | Y N A R G G | CCCTGGCCGCATGCAAAT | P W P H A N | 1 | |
| 166 | L5-154 | TATTCTGATTCTGGGGGT | Y S D S G G | TATGTGTGGCCGTTTCCC | Y V W P F P | 1 | |
