# Supplementary material for: Selection of scFv Antibody Fragments Binding to Human Blood versus Lymphatic Endothelial Surface Antigens by Direct Cell Phage Display
Source: PLoS One. 2015 May 20;10(5):e0127169. doi: 10.1371/journal.pone.0127169 (PMC4439027; doi:10.1371/journal.pone.0127169)
Supplement: S3 Table — a Amino acid lengths of retrieved VH and VL CDR3 sequences; b Occurence of CDR3s with respective amino acid lengths in 166 retrieved diverse scFv antibody clones (for detailed sequences see S2 Table). (DOCX) [file pone.0127169.s013.docx]

**S3 Table**

| **VH CDR3 amino acid length ^a^** | **Occurrence in diverse scFvs ^b^** | **VL CDR3 amino acid length ^a^** | **Occurrence in**  **diverse scFvs ^b^** |
| --- | --- | --- | --- |
| 4 | 8 | 4 | 1 |
| 5 | 10 | 5 | - |
| 6 | 133 | 6 | 156 |
| **Sum** | **151** | **Sum** | **157** |
